# Supplementary material for: Effect of β2-agonist treatment on insulin-stimulated peripheral glucose disposal in healthy men in a randomised placebo-controlled trial
Source: Nat Commun. 2023 Jan 12;14:173. doi: 10.1038/s41467-023-35798-5 (PMC9835033; doi:10.1038/s41467-023-35798-5)
Supplement: Supplementary file 1 — Supplementary Information [file 41467_2023_35798_MOESM1_ESM.pdf]

Supplemental Information

Effect of β2-agonist treatment on insulin-stimulated peripheral glucose disposal in healthy men in a randomised placebo-controlled trial

Sten M.M. van Beek, Yvonne M.H. Bruls, Froukje Vanweert, Ciarán E. Fealy, Niels J. Connell, Gert Schaart, Esther Kornips, Johanna A. Jörgensen, Frédéric M. Vaz, Ellen T.H.C. Smeets, Peter J. Joris, Anne Gemmink, Riekelt H. Houtkooper, Matthijs K.C. Hesselink, Tore Bengtsson, Bas Havekes, Patrick Schrauwen, Joris Hoeks

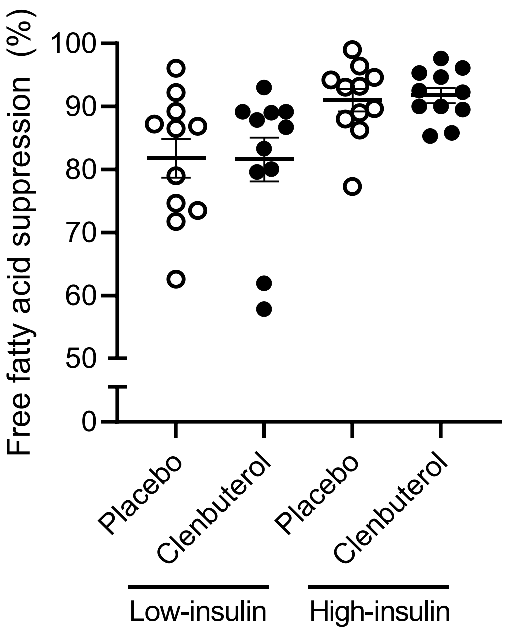

Supplemental Figure 1. *Effect of prolonged clenbuterol treatment on adipose tissue sensitivity expressed as the percentage free fatty acid suppression during both the low- and high- insulin phases of the two-step hyperinsulinemic-euglycemic clamp as compared to baseline. All data as analysed by means of a two-sided Wilcoxon signed-rank test. Placebo: n = 11 per group, Clenbuterol: n = 11 per group. Data are presented as mean ± SEM. Source data are provided as a Source Data file.*

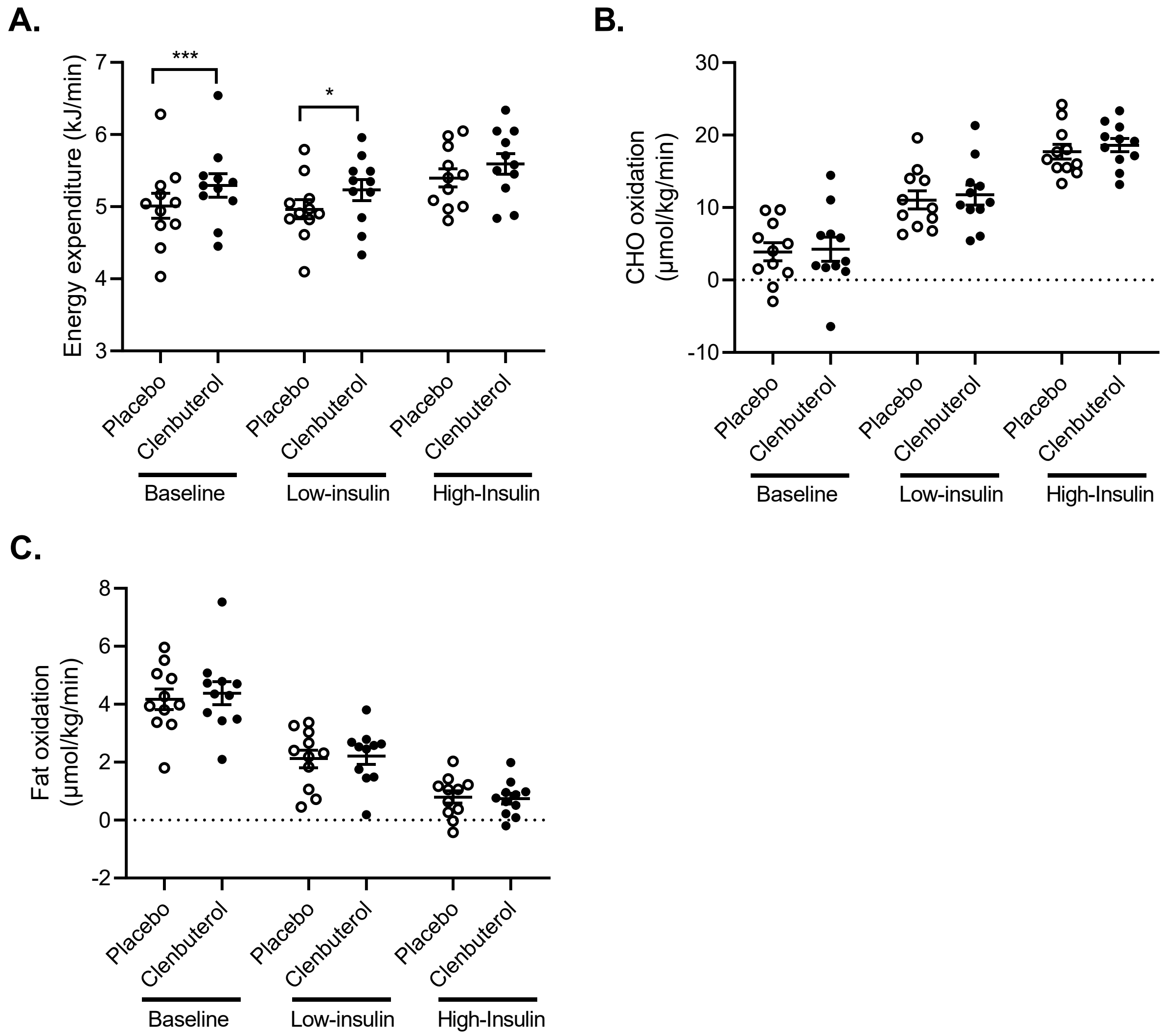

Supplemental Figure 2. *Effect of prolonged clenbuterol treatment on basal metabolic rate and substrate oxidation. A. Energy expenditure during baseline, low- and high-insulin infusion (kJ/min) (Baseline: p < 0.001, low-insulin: p = 0.019). B. Carbohydrate (CHO) oxidation during baseline, low- and high-insulin infusion (µmol/kg/min). C. Fat oxidation during baseline, low- and high-insulin infusion (µmol/kg/min). All data were analysed by means of a two-sided Wilcoxon signed-rank test. Placebo: n = 11 per group, Clenbuterol: n = 11 per group. * p < 0.05, *** p < 0.001. Data are presented as mean ± SEM. Source data are provided as a Source Data file.*

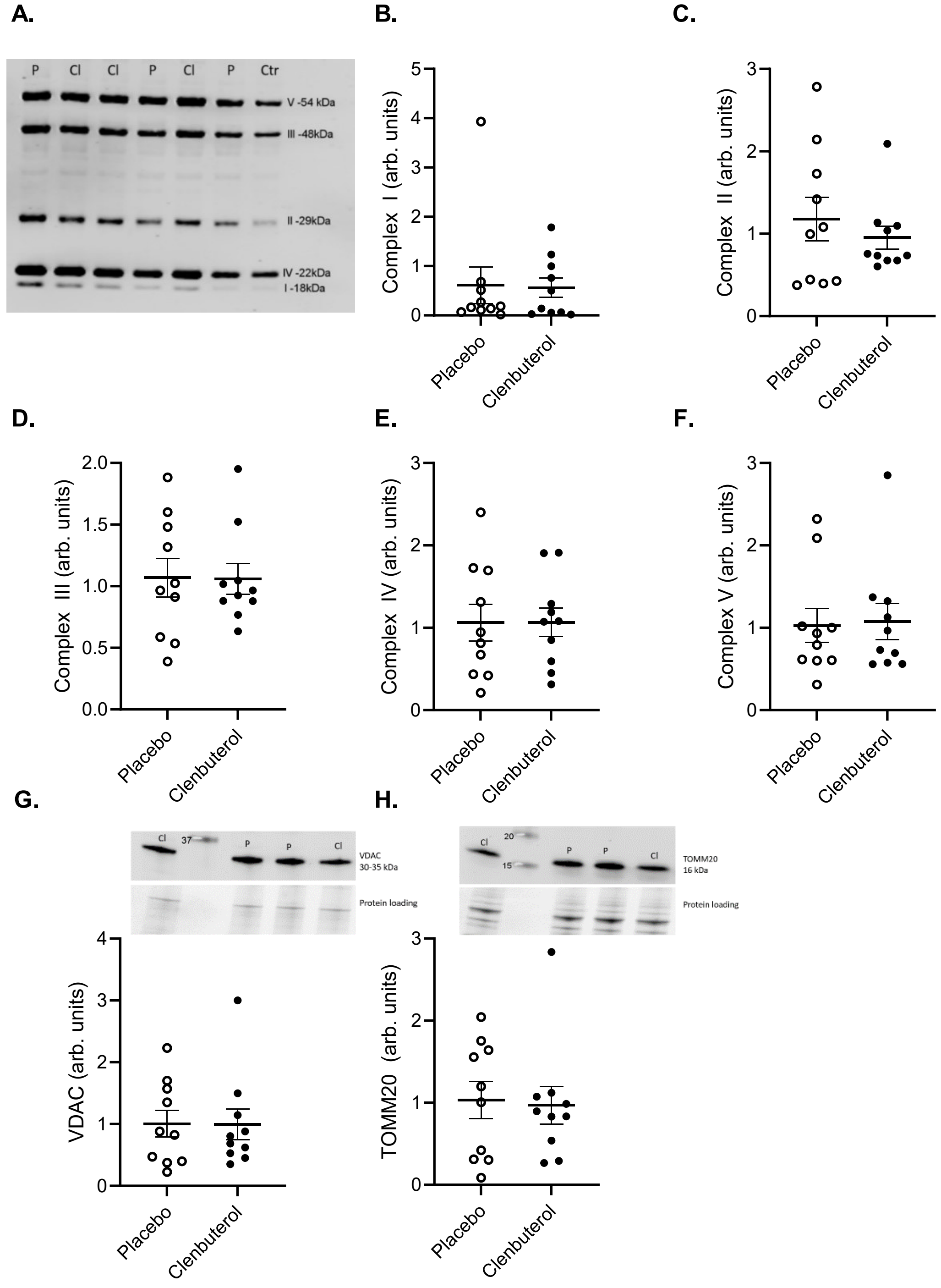

Supplementary Figure 3. *Effect of clenbuterol treatment on markers of mitochondrial function and turnover. A. Representative blot of OXPHOS protein expression. B. Mitochondrial Complex I expression. C. Mitochondrial Complex II expression. D. Mitochondrial Complex III expression. E. Mitochondrial IV expression. F. Mitochondrial Complex V expression. G. Voltage dependent anion channel (VDAC) expression. H. Translocase of outer mitochondrial membrane 20 (TOMM20) expression. Both samples of a participant were run on the same blot for comparison All data were analysed by means of a two-sided Wilcoxon signed-rank test. VDAC = Voltage dependent anion channel, TOMM20 = Translocase of outer mitochondrial membrane 20, P = placebo, Cl = Clenbuterol, Ctr = control. Placebo: n = 10 per group, Clenbuterol: n = 10 per group. Data are presented as mean ± SEM. Source data are provided as a Source Data file.*

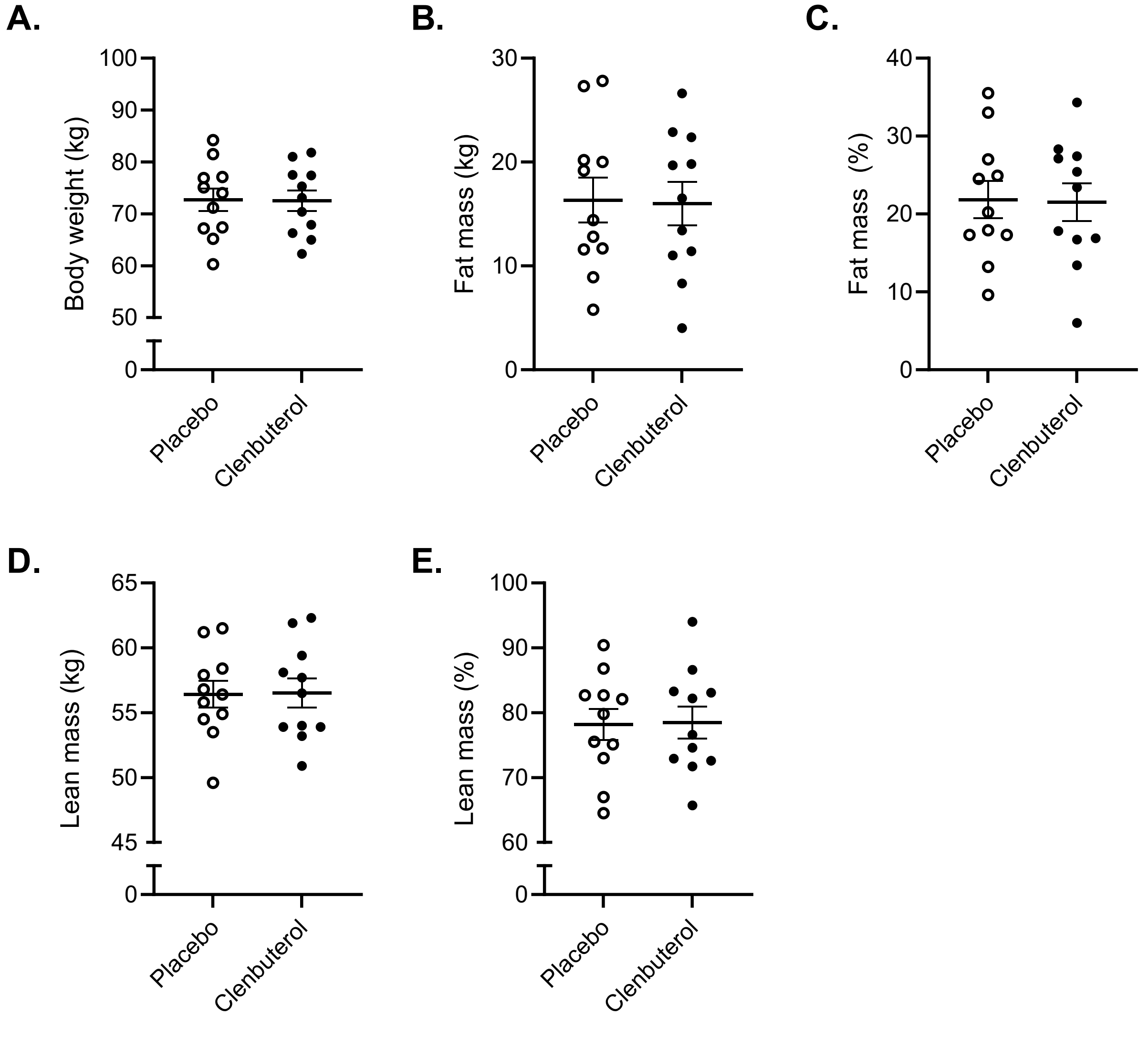

Supplementary Figure 4. *Prolonged clenbuterol administration does not affect body weight or composition. A. Body weight (kg). B. Fat mass (kg). C. Fat mass as percentage of body weight (%). D. Lean mass (kg). E. Lean mass as percentage of body weight (%). All data were analysed by means of a two-sided Wilcoxon signed-rank test. Clenbuterol: n = 11. Placebo: n = 11. Data are presented as mean ± SEM. Source data are provided as a Source Data file.*

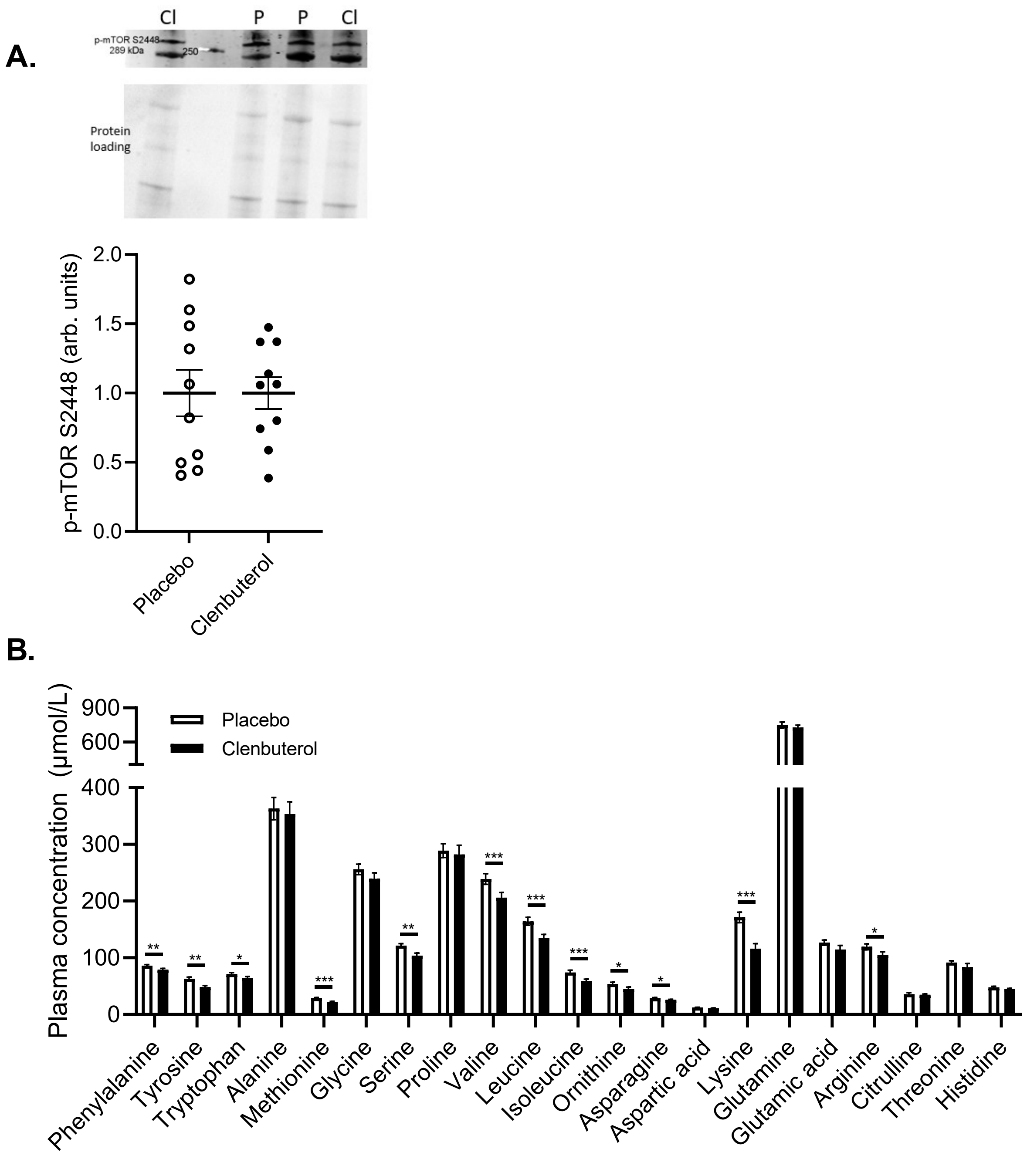

Supplementary Figure 5. *Effect of two-weeks clenbuterol treatment on the activation of mTORC1 and plasma amino acids. A. phosphorylation of mTOR S2448 (mTORC1), B. plasma amino acid concentrations (Phenylalanine: $p = 0.003$, Tyrosine: $p = 0.005$, Tryptophan: $p = 0.042$, Methionine: $p < 0.001$, Serine: $p = 0.005$, Valine: $p < 0.001$, Leucine: $p < 0.001$, Isoleucine $p < 0.001$, Ornithine: $p = 0.014$, Asparagine: $p = 0.019$, Lysine: $p < 0.001$, Arginine: $p = 0.014$). Both samples of a participant were run on the same blot for comparison All data were analysed by means of a two-sided Wilcoxon signed-rank test. P = placebo, Cl = clenbuterol. For mTORC1 n = 10 per group and for plasma amino acids n = 11 per group. * $p < 0.05$, ** $p < 0.01$, *** $p < 0.001$. Data are presented as mean ± SEM. Source data are provided as a Source Data file.*

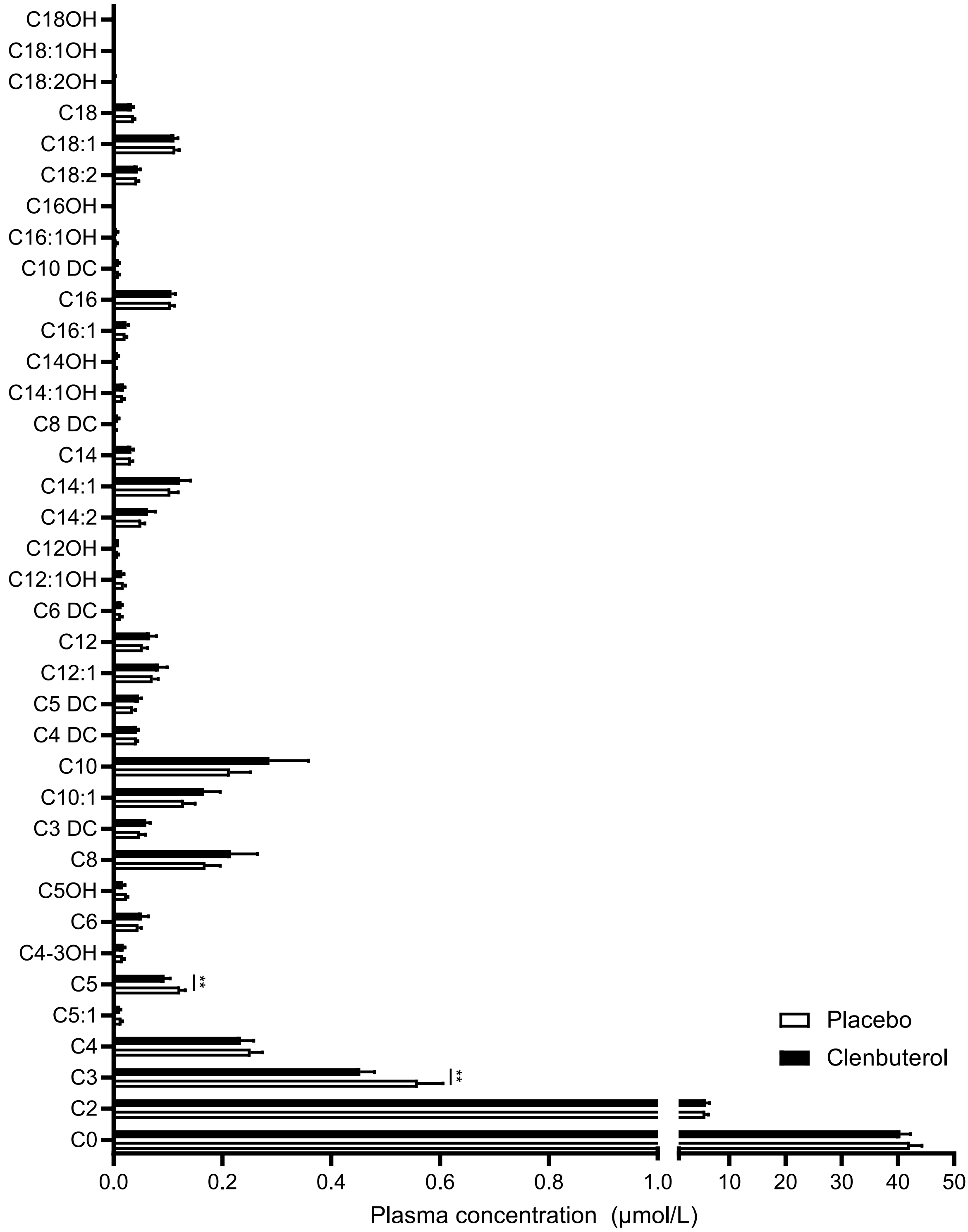

Supplementary Figure 6. *The effect of clenbuterol treatment on fasting plasma acylcarnitine profiles. All data were analysed by means of a two-sided Wilcoxon signed-rank test (C5: p = 0.002, C3: p = 0.007. ** p < 0.01). N = 11 per group. Data are presented as mean ± SEM. Source data are provided as a Source Data file.*

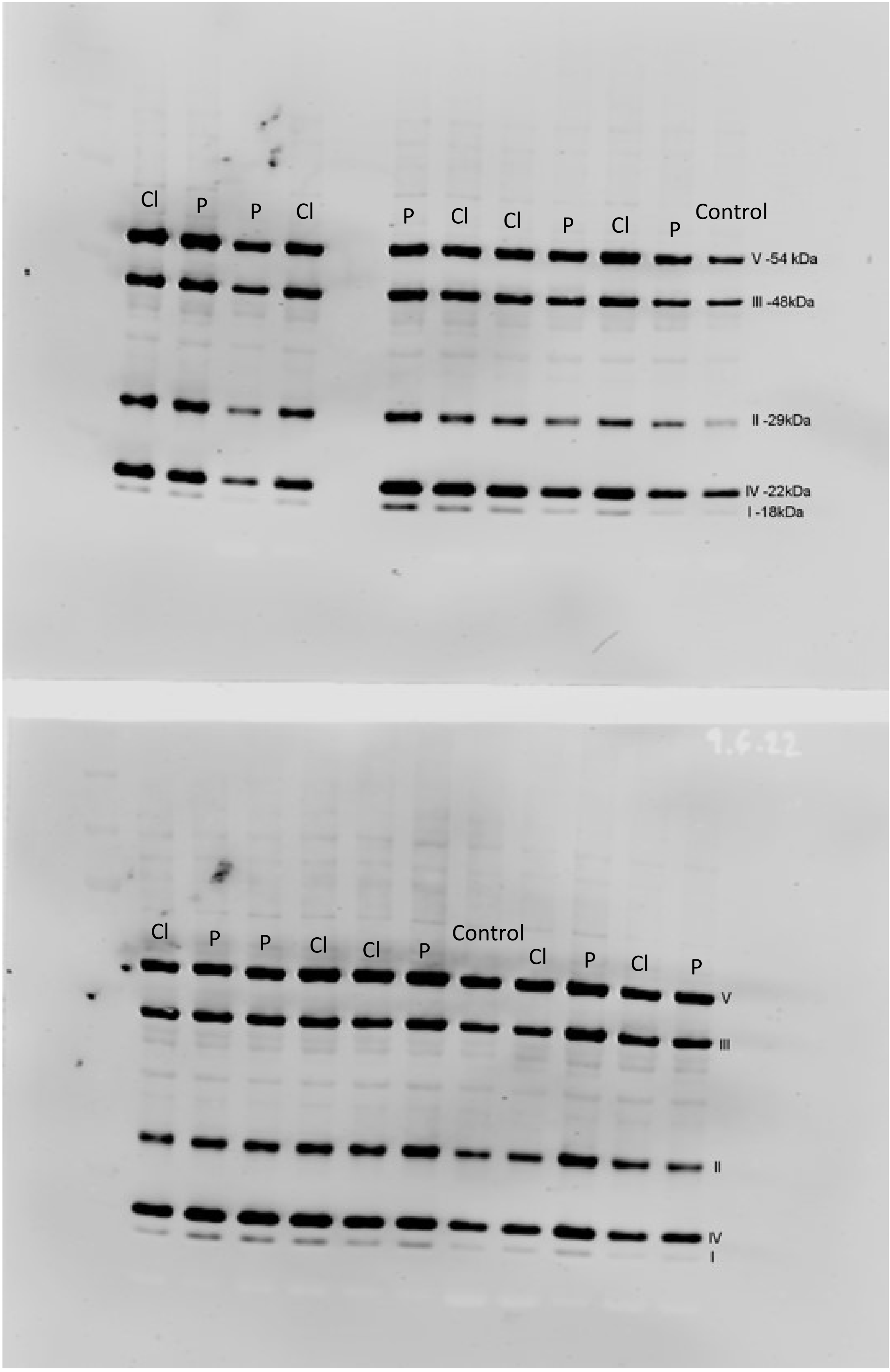

Full western blot Suppl. Fig. 3A-F (OXPHOS complexes). Cl = clenbuterol, P = placebo. Control = tissue from human muscle biopsy.

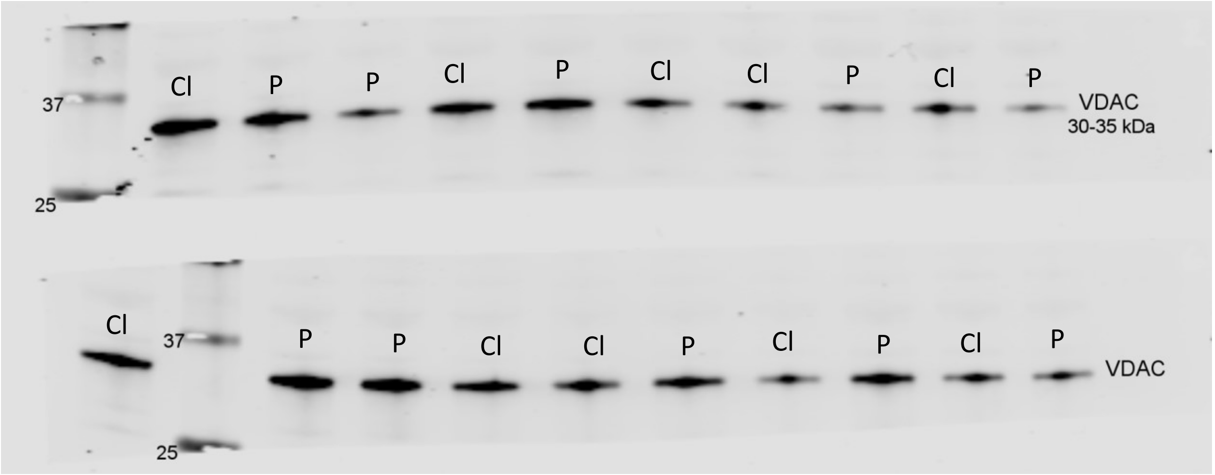

Full western blot Suppl. Fig. 3G (VDAC). Cl = clenbuterol, P = placebo.

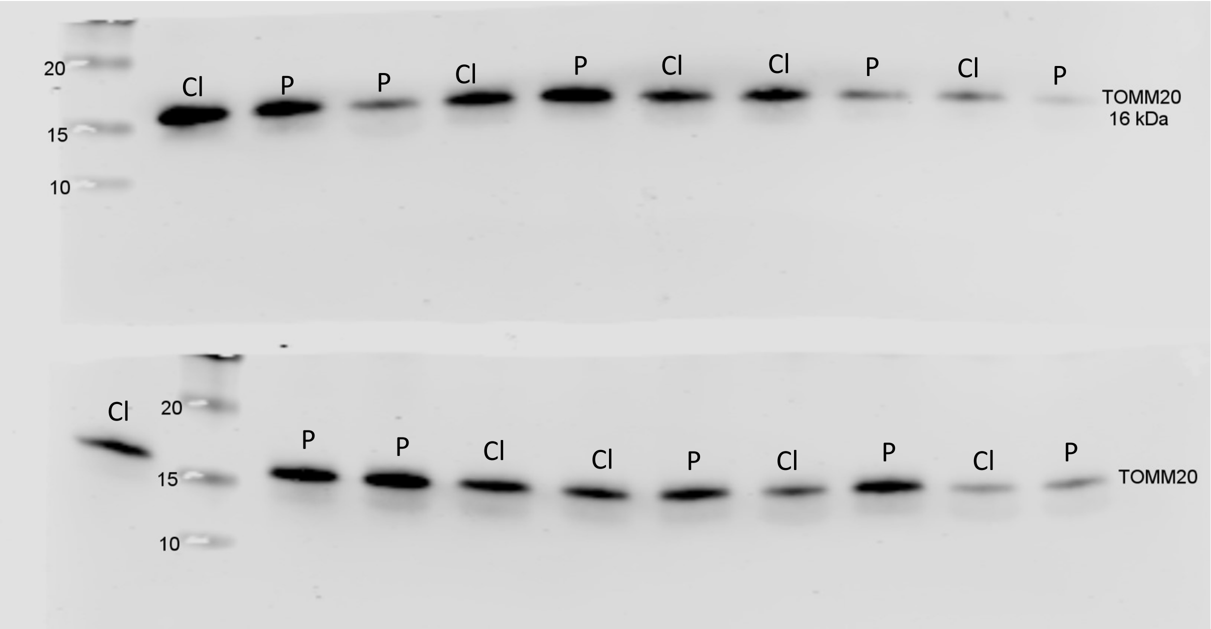

Full western blot Suppl. Fig. 3H (TOMM20). Cl = clenbuterol, P = placebo

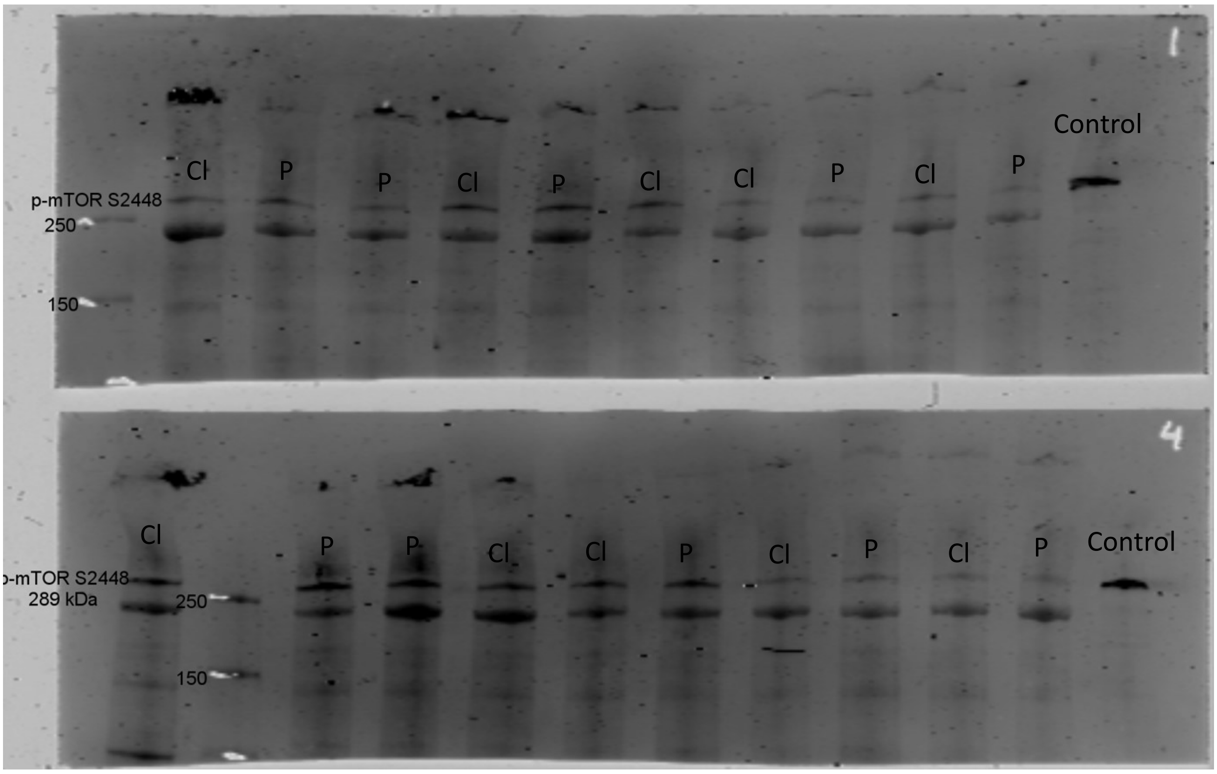

Full western blot Suppl. Fig. 5A (p-mTOR S2448). Cl = clenbuterol, P = placebo. Control consist of C2C12 cells treated with insulin.

Supplementary Note 1

RESEARCH PROTOCOL

Targeting the β_2-adrenergic pathway to improve skeletal muscle glucose uptake in healthy humans

Investigators:

S.M.M. van Beek, MSc, Dept of Nutrition and Movement Sciences, Maastricht University, Maastricht, The Netherlands

Dr. J. Hoeks, Dept of Nutrition and Movement Sciences, Maastricht university, Maastricht, The Netherlands

(June 2019)

PROTOCOL TITLE 'Targeting the β_2-adrenergic pathway to improve skeletal muscle glucose uptake in healthy humans'

**Protocol ID**	Beta-2 study
**Short title**	Human β_2-adrenergic stimulation and muscle glucose uptake
**EudraCT number**	2018-004245-16
**Version**	4
**Date**	21-06-2019
**Coordinating investigator/project leader**	Sten van Beek<br>Universiteitssingel 50 (room G2.254)<br>6229 ER Maastricht<br>Tel – 043 3884254<br>E-mail: Sten.vanbeek@maastrichtuniversity.nl
**Principal investigator(s) (in Dutch: hoofdonderzoeker/ uitvoerder)**	Dr. Joris Hoeks<br>Universiteitssingel 50 (room 0.338)<br>6229 ER Maastricht<br>Tel – 043 3881507<br>E-mail: J.hoeks@maastrichtuniversity.nl
**Sponsor (in Dutch: verrichter/opdrachtgever)**	School of Nutrition and Translational Research in Metabolism (NUTRIM)<br>Maastricht University<br>PO Box 616, 6200 MD Maastricht
**Subsidising party**	ZonMW & the Dutch Diabetes Research Foundation
**Independent expert (s)**	Ronald Henry, MD PhD<br>Maastricht UMC+<br>Interne geneeskunde<br>Tel - 043-3871562<br>E-mail: rma.henry@mumc.nl

**Laboratory sites**	Maastricht University<br>Department of Nutrition and Movement Sciences<br>Universiteitssingel 50, 6229 ER, Maastricht<br>Metabolic research unit (MRUM)<br><br>Maastricht UMC+<br>Centraal Diagnostisch Laboratorium<br>PO Box 5800, 6202 AZ Maastricht
**Pharmacy**	Apotheek Radboud Universitair Medisch Centrum<br>Postbus 9101, 6500 HB Nijmegen<br><br>Apotheek Maastricht UMC+, P. Debyelaan 25<br>6202 AZ Maastricht

PROTOCOL SIGNATURE SHEET

Name	Signature	Date
Head of Department		
[Coordinating Investigator/Project lea-der/Principal Investigator]:		

TABLE OF CONTENTS

1. INTRODUCTION AND RATIONALE 22
2. OBJECTIVES 25
3. STUDY DESIGN 26
4. STUDY POPULATION 30
   4.1 Population (base) 30
   4.2 Inclusion criteria 30
   4.3 Exclusion criteria 30
   4.4 Sample size calculation 31
5. TREATMENT OF SUBJECTS 33
   5.1 Investigational product/treatment 33
   5.2 Use of co-intervention (if applicable) 33
   5.3 Escape medication (if applicable) 33
6. INVESTIGATIONAL PRODUCT 34
   6.1 Name and description of investigational product(s) 34
   6.2 Summary of findings from non-clinical studies 35
   6.3 Summary of findings from clinical studies 35
   6.4 Summary of known and potential risks and benefits 35
   6.5 Description and justification of route of administration and dosage 36
   6.6 Dosages, dosage modifications and method of administration 36
   6.7 Preparation and labelling of Investigational Medicinal Product 36
   6.8 Drug accountability 37
7. NON-INVESTIGATIONAL PRODUCT 38
   7.1 Name and description of non-investigational product(s) 38
   7.2 Summary of findings from non-clinical studies 38
   7.3 Summary of findings from clinical studies 38
   7.4 Summary of known and potential risks and benefits 38
   7.5 Description and justification of route of administration and dosage 39
   7.6 Dosages, dosage modifications and method of administration 39
   7.7 Preparation and labelling of Non Investigational Medicinal Product 40
   7.8 Drug accountability 40
8. METHODS 41
   8.1 Study parameters/endpoints 41
      8.1.1 Main study parameter/endpoint 41
      8.1.2 Secondary study parameters/endpoints (if applicable) 41
      8.1.3 Other study parameters (if applicable) 41
   8.2 Randomisation, blinding and treatment allocation 41
   8.3 Study procedures 42
   8.4 Withdrawal of individual subjects 47
      8.4.1 Specific criteria for withdrawal (if applicable) 47
   8.5 Replacement of individual subjects after withdrawal 48

8.6 Follow-up of subjects withdrawn from treatment 48
8.7 Premature termination of the study 48
9. SAFETY REPORTING 49
9.1 Temporary halt for reasons of subject safety 49
9.2 AEs, SAEs and SUSARs 49
9.2.1. Adverse events (AEs) 49
9.2.2. Serious adverse events (SAEs) 49
9.2.3. Suspected unexpected serious adverse reactions (SUSARs) 50
9.3. Annual safety report 51
9.4. Follow-up of adverse events 51
9.5. [Data Safety Monitoring Board (DSMB) / Safety Committee] 51
10. STATISTICAL ANALYSIS 52
10.2. Primary study parameter(s) 52
10.3. Secondary study parameter(s) 53
10.4. Other study parameters 53
10.5. Interim analysis (if applicable) 53
Not applicable 53
11. ETHICAL CONSIDERATIONS 54
11.2. Regulation statement 54
11.3. Recruitment and consent 54
11.4. Objection by minors or incapacitated subjects (if applicable) 54
11.5. Benefits and risks assessment, group relatedness 55
11.6. Compensation for injury 56
11.7. Incentives (if applicable) 57
12. ADMINISTRATIVE ASPECTS, MONITORING AND PUBLICATION 58
12.2. Handling and storage of data and documents 58
12.3. Monitoring and Quality Assurance 58
12.4. Amendments 59
12.5. Annual progress report 59
12.6. Temporary halt and (prematurely) end of study report 59
12.7. Public disclosure and publication policy 60
13. STRUCTURED RISK ANALYSIS 61
13.2. Potential issues of concern 61
13.3. Synthesis 63
14. REFERENCES 64

LIST OF ABBREVIATIONS AND RELEVANT DEFINITIONS

**ABR**	**ABR form, General Assessment and Registration form, is the application form that is required for submission to the accredited Ethics Committee (In Dutch, ABR = Algemene Beoordeling en Registratie)**
**AE**	**Adverse Event**
**AMPK**	**AMP-activated protein kinase**
**AR**	**Adverse Reaction**
**β-ARs**	**β-adrenergic receptors**
**ALAT**	**Alanine aminotransferase**
**ASAT**	**Aspartate transaminase**
**BMI**	**Body mass index**
**CA**	**Competent Authority**
**cAMP**	**Cyclic adenosine monophosphate**
**CCMO**	**Central Committee on Research Involving Human Subjects; in Dutch: Centrale Commissie Mensgebonden Onderzoek**
**CV**	**Curriculum Vitae**
**DSMB**	**Data Safety Monitoring Board**
**eCRF**	**Electronic case report form**
**ELISA**	**Enzyme-linked immunosorbent assay**
**EU**	**European Union**
**EudraCT**	**European drug regulatory affairs Clinical Trials**
**Gamma-GT**	**Gamma-glutamyltransferase**
**GCP**	**Good Clinical Practice**
**GLUT4**	**Glucose transporter 4**
**HDL**	**High-density lipoprotein**

**IB**	**Investigator's Brochure**
**IC**	**Informed Consent**
**IMP**	**Investigational Medicinal Product**
**IMPD**	**Investigational Medicinal Product Dossier**
**LDL**	**Low-density lipoprotein**
**METC**	**Medical research ethics committee (MREC); in Dutch: medisch ethische toetsing commissie (METC)**
**MRUM**	**Metabolic research centre at Maastricht University**
**mTOR**	**Mammalian target of rapamycin**
**mTORC**	**Mammalian target of rapamycin complex 2**
**NSAIDs**	**Nonsteroidal anti-inflammatory drugs**
**Rd**	**Glucose disposal rate**
**RT-qPCR**	**Real-time quantitative polymerase chain reaction**
**(S)AE**	**(Serious) Adverse Event**
**SPC**	**Summary of Product Characteristics (in Dutch: officiële productinfomatie IB1-tekst)**
**Sponsor**	**The sponsor is the party that commissions the organisation or performance of the research, for example a pharmaceutical company, academic hospital, scientific organisation or investigator. A party that provides funding for a study but does not commission it is not regarded as the sponsor, but referred to as a subsidising party.**
**SUSAR**	**Suspected Unexpected Serious Adverse Reaction**
**T2DM**	**Type 2 diabetes mellitus**
**Wbp**	**Personal Data Protection Act (in Dutch: Wet Bescherming Persoonsgevens)**
**WMO**	**Medical Research Involving Human Subjects Act (in Dutch: Wet Medisch-wetenschappelijk Onderzoek met Mensen**

SUMMARY

Rationale: Type 2 diabetes mellitus (T2DM) and its associated cardiovascular comorbidities have developed into a leading cause of death in western countries. Medical and non-medical treatments have failed to counter this 'diabesity' epidemic, fostering the need for novel therapies. In this context, we have recently demonstrated robust improvements in insulin sensitivity in T2DM patients upon 10 days of mild cold acclimatisation, which proved to be primarily mediated through an increased skeletal muscle glucose uptake and occurred independent of improvements in classical regulatory pathways (i.e. insulin signalling or AMPK activation). Given this background, it was recently shown that skeletal muscle glucose uptake can also be mediated through an alternative novel pathway involving β_2-adrenergic receptors, through activation of mTORC2. Thus, animal studies showed robust improvements in glucose homeostasis in diabetic rodents upon prolonged treatment with a low-dose of the selective β_2-agonist clenbuterol. This project aims to investigate the human relevance of this novel β_2-mTORC2 pathway and intents to investigate if prolonged supplementation with the selective β_2-adrenergic agonist clenbuterol improves glucose disposal in healthy, lean male individuals. As such, this study serves to identify if this pathway could potentially be used as a novel treatment target to improve glucose homeostasis T2DM patients.

Objective: The primary research objective is the change in the insulin-stimulated peripheral glucose disposal rate (Rd), expressed as (μmol/kg/min) upon 2 weeks clenbuterol vs. placebo supplementation. The secondary objective is the change in skeletal muscle GLUT4 translocation.

Study design: 2-week randomized, double-blinded, placebo-controlled, cross-over design with a 4-week wash-out period.

Study population: 23 healthy, lean male participants (BMI: 20-25 kg/m^2) aged between 18-30 years.

Intervention (if applicable): 2-week oral supplementation with clenbuterol (40 μg/day) or placebo. Capsules (20 μg) will be consumed twice daily.

Main study parameters/endpoints: Main study parameter is insulin-stimulated peripheral glucose disposal (Rd) during the high-insulin infusion rate during the two-step hyperinsulinemic-euglycemic clamp. The secondary endpoint is skeletal muscle GLUT4 translocation. This is performed by means of immunohistochemistry and assessed by wide-field microscopy.

Nature and extent of the burden and risks associated with participation, benefit and group relatedness: During this study we will investigate the effect of prolonged β_2-adrenergic

agonist supplementation on skeletal muscle glucose uptake and insulin sensitivity in healthy male adults. This research could potentially lead to the development of novel strategies to treat T2DM. This study will not induce any benefits for the subjects and the major burden will be the time investment and potential side effects of clenbuterol. In total, the subjects will visit the University of Maastricht on 6 occasions (excluding screening) for measurements. Performed measurements will be without risks, but hematomas or bruises could develop upon blood sampling or muscle biopsies taken. This risk will be minimized due to state-of-the-art techniques and sterility measures taken. Clenbuterol or placebo supplementation will be given for 14 days, in which subjects ingest 1 capsule (20 μg) twice daily (40 μg/day). Clenbuterol could induce adverse effects, e.g. headache, increased heart rate/blood pressure, tremors, dizziness. To minimize the risks of adverse events, we deliberately choose to perform the study in a young, healthy population, using a standard dose clenbuterol (40 μg/day) as well as a short treatment duration. To limit the number of subjects that need to be included we decided for a cross-over design in which every participant serves as his own control.

1. INTRODUCTION AND RATIONALE

Introduction

In modern society, the prevalence of obesity has reached epidemic proportions (1). Importantly, obesity is highly associated with the development of type 2 diabetes mellitus (T2DM) and several other comorbidities (1). In fact, mainly due to this epidemic prevalence of obesity, T2DM and its associated cardiovascular diseases have emerged as one of the leading causes of death in Western countries, annually accounting for over 3.5 million deaths according to the World Health Organization (2). Non-medical treatment strategies to counter this 'diabesity' epidemic, including healthy dietary habits and physical activity, are primarily focussed on reducing extensive energy excess in the human body. Whilst highly effective, long-term adherence to exercise training programs and dietary regimes is poor, fostering the need for novel therapies.

In search for alternative ways to beneficially affect glucose homeostasis, we have investigated the effects of prolonged, intermittent mild cold exposure (3, 4). Although these mild cold acclimation studies were primarily focussed on the activation of brown adipose tissue, we demonstrated that mild cold exposure (15-16℃) for 10 consecutive days (6h/day) significantly enhanced GLUT4 translocation in skeletal muscle in T2DM patients (5). Remarkably, these observed effects on skeletal muscle GLUT4 were paralleled by a robust ~43% improvement in peripheral insulin stimulated glucose uptake, an effect that even exceeds the effects of prolonged exercise training (6), generally accepted as one of the most efficient therapies to prevent and treat T2DM (5, 7). Interestingly, the previously mentioned GLUT4 translocation upon the mild cold acclimation in T2DM patients appeared to occur independently from both the activation of key proteins of the insulin signalling pathway and adenosine monophosphate (AMP)-activated protein kinase (AMPK) (5), an important player in contraction-mediated GLUT4 translocation (8). Based on these collective findings, we speculated that alternative pathways bypassing the conventional insulin signalling and AMPK pathways are responsible for the increased skeletal muscle glucose uptake upon mild cold exposure.

In light of this, a research group at Stockholm University has uncovered a novel pathway mediating skeletal muscle glucose uptake through the activation of β_2-adrenergic receptors (β_2-ARs), the major subtype of β-ARs in myocytes (9-11). In more detail, activation of β_2-ARs markedly increased GLUT4-mediated glucose uptake in both L6 muscle cells and human primary myotubes through the activation of the mammalian target of rapamycin (mTOR) complex 2 (mTORC2) (**Figure 1**). Importantly, the β_2-AR mediated increases in

skeletal muscle glucose uptake occurred independent of the activation of Akt, AS160 and AMPK (9), similar to the findings of our cold acclimation study in T2DM patients (5). In addition, this pathway was shown to be physiologically relevant, meaning that the activation of β_2-ARs induced glucose uptake into skeletal muscle both *ex vivo* and *in vivo*, and these effects were abolished in β_1/β_2- AR knockout mice (9). Since cold-exposure is well-known to activate the sympathetic nervous system (12), this novel pathway hence provides a likely mechanism to explain the beneficial effects of cold acclimation on skeletal muscle glucose disposal.

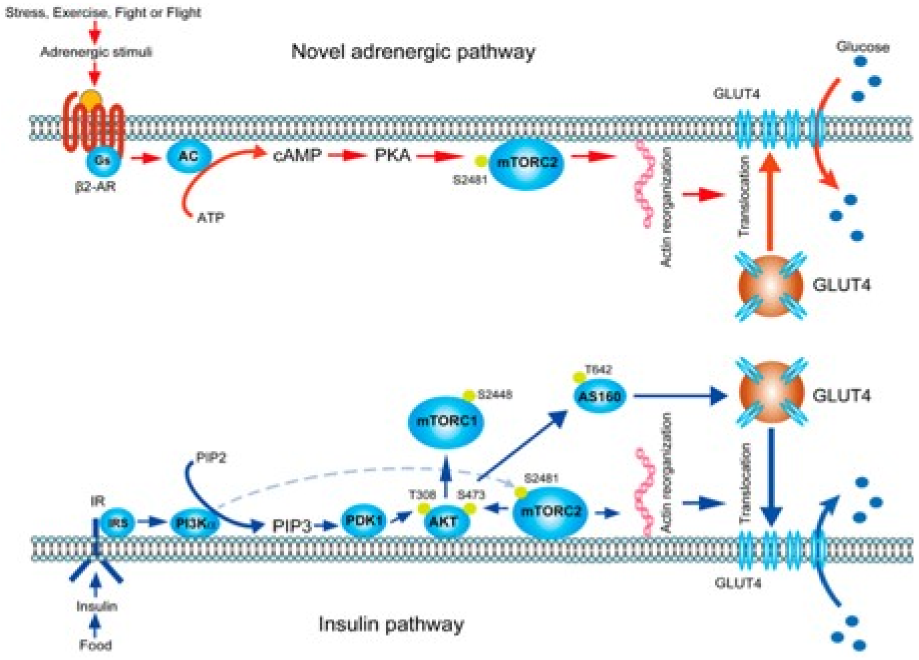

Figure 1: novel, insulin-independent pathway to stimulate glucose uptake in skeletal muscle. *The β_2-adrenoceptor pathway, when activated, couples to G_S to stimulate adenylate cyclase (AC), resulting in the production of cAMP, activation of PKA and phosphorylation of mTORC2. The phosphorylated mTORC2 then stimulates actin reorganization and GLUT4 translocation to the plasma membrane to increase glucose uptake. The lower part of the graph represents the classical insulin signaling pathway. From ref. (Sato et al. 2014).*

The existence of a novel pathway mediating skeletal muscle glucose disposal, independent of the activation of the insulin and AMPK pathway, presents an interesting novel target to improve glucose disposal in T2DM patients. Indeed, the lab in Stockholm showed that

short-term supplementation (4 days) with a relatively high dosage (30 mg/L in the drinking water) of clenbuterol, a selective β_2-adrenergic agonist, dramatically improved glucose tolerance in both Goto/Kakazaki rats and high-fat diet-induced (DIO) mice, two well-established animal models for diabetes (9). Furthermore, studies performed by Castle et al. (13) and Pan et al. (14) showed similar improvements in glucose homeostasis in obese Zucker rats upon prolonged supplementation with a relatively high-dose of clenbuterol. Evidently, however, high dosages of clenbuterol are strongly associated with detrimental side effects in humans, resulting in an increased heart rate/blood pressure, muscle spasms and tremors (15), which impairs translation towards clinical application. In line of this, recent work performed by our collaborators in Stockholm has demonstrated that the activation of the β_2-mTORC2 pathway through supplementation with a low-dose of clenbuterol still significantly induced beneficial effects on glucose homeostasis in DIO mice.

Taken together, we have compelling evidence that (even a low dose of) the selective β_2-agonist clenbuterol robustly improves whole-body glucose homeostasis *in vivo* in mice via the β_2-mTORC2 pathway. However, whether the β_2-mTORC2 pathway can be activated in humans *in vivo* to improve glucose disposal, has thus far not been investigated.

Hypothesis

Here, we hypothesize that activation of the novel mTORC2 pathway via selective β_2-adrenergic stimulation increases skeletal muscle glucose uptake and beneficially affects metabolic health in healthy lean male volunteers with normal physical activity.

2. OBJECTIVES

Primary Objective:

- To determine if prolonged treatment with the selective β_2-adrenergic agonist clenbuterol improves glucose disposal via the mTORC2 pathway in lean, healthy male individuals with normal physical activity.

Secondary Objective(s):

- Does acute (4 hours) administration with the selective β_2-adrenergic agonist clenbuterol enhance skeletal muscle GLUT4 translocation via the mTORC2 pathway?
- Does prolonged (2 weeks) administration with the selective β_2-adrenergic agonist clenbuterol enhance skeletal muscle GLUT4 translocation via the mTORC2 pathway?

Explorative objectives:

- Does acute (4 hours) and prolonged (2 weeks) administration with the selective β_2-adrenergic agonist clenbuterol affect:
  - Body weight/composition
  - Plasma substrates
  - Heart rate and blood pressure
  - Insulin-mediated suppression of hepatic glucose production
  - (Sleeping) energy expenditure and substrate oxidation
  - Skeletal muscle glycogen and lipid content
  - Gene and protein expression in skeletal muscle
  - Femoral artery flow mediated dilation (FMD)

3. STUDY DESIGN

To investigate the effects of prolonged treatment with a selective β_2- agonist on whole-body glucose disposal, a clinical trial will be conducted with 16 young, healthy, lean male subjects. Thus, in a randomized, placebo-controlled, double-blinded, cross-over design, subjects will receive either the selective β_2- agonist clenbuterol (40 μg/day) or a placebo for 2 weeks with a 4-week wash-out period **(Figure 2)**. The study ends when 11 participants completed the second intervention period.

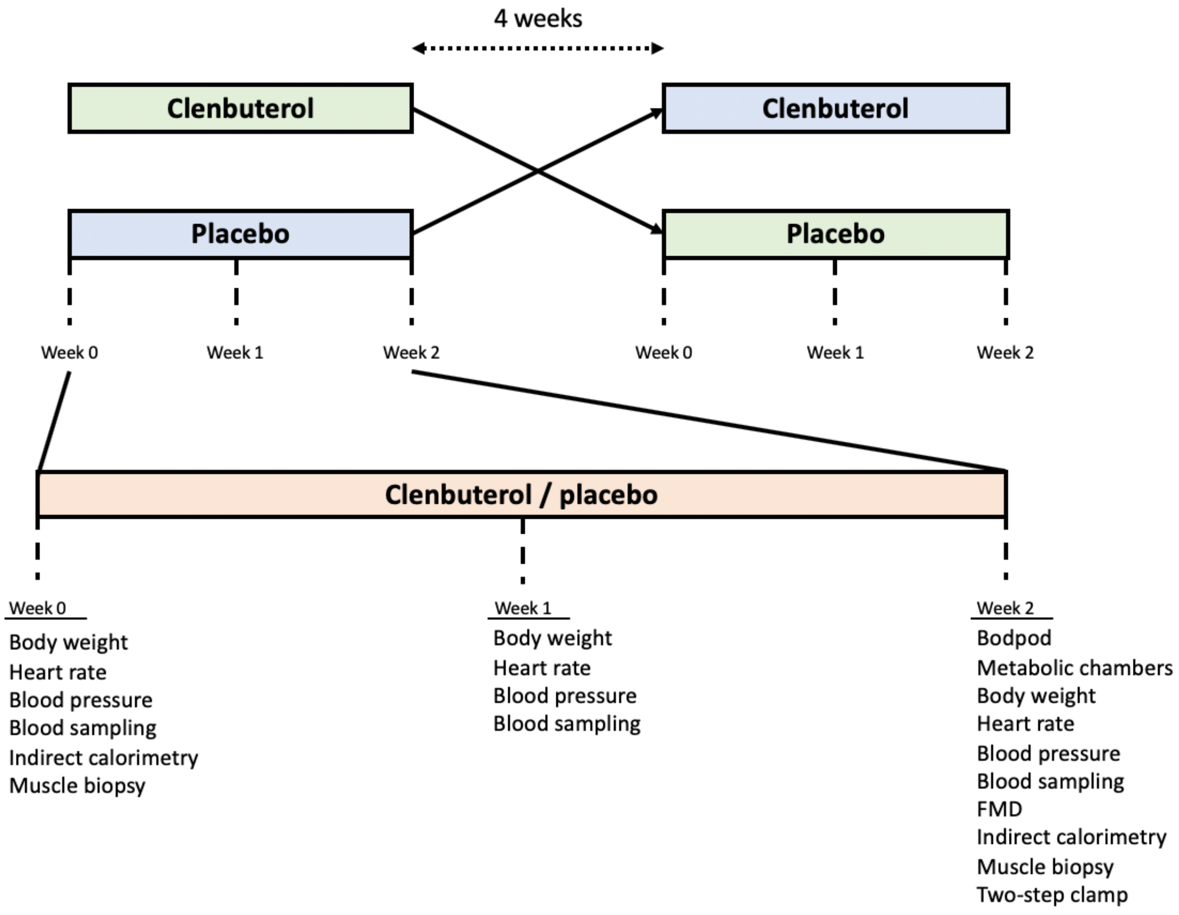

Figure 2 Study design. *A randomized, placebo-controlled, single-blinded, cross-over design, in which subjects will receive either daily clenbuterol (40 μg/day) or placebo supplementation for 2 weeks with a 4-week wash-out period. Participants will be randomly allocated to one of the study arms. Several metabolic read-out parameters will be evaluated upon treatment. Acute effects of clenbuterol on skeletal muscle metabolism will be investigated with indirect calorimetry, with regular blood sampling, 4 hours after the consumption of the first dose of each study arm, after which a muscle biopsy will be taken. After 14 days of supplementation, Bodpod measurements will be performed to assess effects on body composition and an FMD measurement will be performed. Following an overnight fast, a muscle biopsy will be taken and a hyperinsulinemic-euglycemic clamp will be performed. All measurements within the first treatment period will be repeated within the second treatment period. A wash-out period of 4-weeks will be applied. All participants will be closely monitored for AEs during the supplementation period.*

For this study, the preferred drug of use is the selective β_2-adrenergic agonist clenbuterol. Clenbuterol can – in contrast to other selective β_2- agonists – be administered orally due to its water-soluble composition and is additionally long acting (T_{50} = 35 hours, (16)), thereby ensuring systemic stimulation throughout the day. To minimize the risk of adverse events and to prevent any confounding effects of changes in body composition that are commonly associated with prolonged treatment with high doses of clenbuterol (17), we deliberately choose to perform the study in a completely healthy population using a well-tolerable, standard dose of 40 μg/day (normal recommended dose for clinical use: 40-80 μg/day) in combination with a short-term treatment duration.

3.1 Detailed study design

After an initial screening, subjects will visit the Metabolic Research Centre at Maastricht University (MRUM) at 6 occasions (3 occasions per study arm) (**for details please see Section 8.3 → 'study procedures'** and Table 1) with a wash-out period of 4-weeks:

Screening *(time investment = 1 hour)*

After the subject has shown interest in the study, a screening will be scheduled to assess whether the subject is suitable for the study. The subject will arrive at the university after an overnight fast. The screening will include filling in a medical history and physical activity questionnaire (See Document F1). Body weight, heart rate and blood pressure will be measured, an ECG will be performed followed by the collection of a fasted blood sample (~15 mL) for determination of clinical health parameters, including markers for kidney and liver function (creatinine ASAT, ALAT and gamma-GT for liver).

Study period

Day 1 *(time investment = 6 hours/study arm)*

At the start of each treatment period, subjects will arrive at the MRUM after an overnight fast (i.e. no food consumption after 22:00 the night before). Body weight, heart rate and blood pressure will be measured, followed by the collection of a fasting blood sample to determine circulating plasma substrate concentrations (~15 mL). Baseline energy expenditure will be measured by means of indirect calorimetry for 30 minutes (Ventilated Hood, Omnical, Maastricht University). Afterwards, subjects will consume their first capsule (clenbuterol or

placebo) under supervision of the researcher. The following 4 hours after initial supplement intake, blood samples will be collected every hour to determine circulating plasma substrate concentrations (as described above). Furthermore, blood pressure and heart rate measurements will be performed hourly. Measurement of energy expenditure and substrate oxidation will be performed by means of indirect calorimetry for 30 minutes every hour. After 4 hours, a muscle biopsy will be collected for the determination of skeletal muscle GLUT4 translocation, lipid content and gene and protein expression. Before subjects go home, they will receive 1 weeks' worth of supplements and will be instructed on the dose, frequency and route of administration of the supplements. Furthermore, the subjects are instructed to get in contact with the researcher in case any side effects emerge.

Day 8 *(time investment = 1 hour/study arm)*

After 1 week of supplementation, subjects will arrive at the MRUM in a fasted state for a half-way safety check-up. Body weight, heart rate and blood pressure will be measured, followed by a fasting blood sample collection for determination of circulation plasma substrate concentrations (~15 mL). Subjects will receive 1 weeks' worth of supplements which will last till the end of the supplementation period.

Day 14

At day 14 of each study arm, subjects will arrive at the MRUM at 17:00 and an FMD measurement is performed by means of an Echo-Doppler. Afterwards, subjects will receive a standardized meal, provided by the researcher. Around 18:00, subjects will enter a respiration chamber where they will stay overnight. This whole-room calorimeter is equipped with a toilet, sink, bed, desk, computer and television. At 11:00 pm, the lights will be turned off and the subject is instructed to try to sleep. The overnight stay in the respiration chamber is performed to measure the subject's sleeping metabolic rate upon clenbuterol/placebo supplementation, as well as to create equal conditions between both study periods prior to the primary outcome, the two-step hyperinsulinemic-euglycemic clamp.

Day 15 *(time investment day 14 and 15 = 26 hours/study arm)*

At day 15 of the supplementation period of each study arm, subjects will exit the respiration chamber in the fasted state and body composition will be assessed by means of the BodPod (Cosmed). Afterwards, blood pressure and heart rate will be measured and a fasted blood

sample will be collected for determination of circulating plasma substrate concentrations (~15 mL). A skeletal muscle biopsy will be taken at baseline for the determination of the effect of prolonged clenbuterol supplementation on GLUT4 translocation, lipid content and gene and protein expression (i.e. mTORC2, insulin signalling, and AMPK pathways). Following the muscle biopsy, a 2-step hyperinsulinemic-euglycemic clamp with indirect calorimetry will be performed for the determination of peripheral and hepatic insulin stimulated glucose disposal.

Table 1. Overview of measurements and time investment

**Visit (day)**	**Effect of clenbuterol**	**Test**	**Time per visit (hours)**	**Total time (hours)**
0		Screening: body weight, heart rate, blood pressure, blood sampling, ECG	1	1
1	Acute	Body weight, heart rate, blood pressure, blood sampling, indirect calorimetry, muscle biopsy	6	12
8	Long-term	Body weight, heart rate, blood pressure and blood sampling	1	2
14	Long-term	FMD, Metabolic chamber		
15	Long-term	Bodpod, Muscle biopsy and two-step hyperinsulinemic-euglycemic clamp	26	52
**Total amount of time investment**			34	67

4. STUDY POPULATION

4.1 Population (base)

The study population will consist of healthy, lean (BMI 20-25 kg/m^2) Caucasian males. The study will be restricted to males to prevent hormonal influences of the menstrual cycle and known gender differences in metabolism. Subjects have to be aged between 18-30 years old. For recruitment procedures see section 8.3.1. Subjects must be able to provide written informed consent, meet all the inclusion criteria and none of the exclusion criteria.

4.2 Inclusion criteria

In order to be eligible to participate in this study, a subject must meet all of the following criteria:

1. Caucasian;
2. Male sex;
3. Age between 18-30 years
4. BMI: 20-25 kg/m^2;
5. Normal physical activity levels;

4.3 Exclusion criteria

A potential subject who meets any of the following criteria will be excluded from participation in this study:

1. Not meeting all inclusion criteria
2. Cardiovascular disease (determined by means of questionnaires, heart rate/blood pressure measurements and an ECG)
3. Respiratory diseases (including asthma, bronchitis and COPD);
4. Unstable body weight (weight gain or loss > 5 kg in the last three months);
5. Intention to lose or gain body weight (e.g. with caloric restriction or physical activity)
6. Excessive alcohol and/or drug abuse;
7. Hypokalaemia;
8. Hyperthyroidism
9. Anaemia;
10. Epilepsy;
11. Smoking;
12. Renal and/or liver insufficiency;

13. Participation in another biomedical study within 1 month before the first study visit, possibly interfering with the study results;
14. Medication use known to hamper subject's safety during the study procedures;
15. Subjects who do not want to be informed about unexpected medical findings;
16. Subjects who do not want that their treating physician to be informed;
17. Inability to participate and/or complete the required measurements;
18. Participation in organised or structured physical exercise;
19. Any condition, disease or abnormal laboratory test result that, in the opinion of the Investigator, would interfere with the study outcome, affect trial participation or put the subject at undue risk;

A medical doctor will judge participation eligibility based on the medical history questionnaire, medication use and fasting blood parameters. If the medical doctor advises that a subject cannot participate, he will be excluded from enrolment.

4.4 Sample size calculation

The primary endpoint of this study is glucose disposal rate (Rd) during the hyperinsulinemic-euglycemic clamp, expressed as μmol/kg/min. As no previous studies have been performed regarding the effect of prolonged clenbuterol supplementation on the glucose homeostasis, standard deviations and estimated effect size are based on an intervention study performed previously by our research group in healthy, lean male subjects (18). Sample size calculation was performed using a paired sample T-test (two-sided):

$$n = \frac{\sigma^2 (z_{\alpha/2} + z_{\pi})^2}{\Delta\mu^2}$$

Based on this paired samples T-test, the expected standard deviation in glucose disposal during a clamp (Rd) in young, lean subjects (9.7 μmol/kg/min) and an expected mean difference of 25%, which is in our opinion a physiological relevant percentage, we calculated that 11 subjects are required to reject the null hypothesis with a probability (power) of 80%. The type 1 error probability (α) is 0.05. Since we anticipate a drop-out rate of about 30%, we assume that a total number of 16 subjects have to be included in the study. Furthermore, it is

estimated that 70% of screened subjects will eventually be included in the study and, therefore, a total of 23 subjects will be screened.

TREATMENT OF SUBJECTS

4.5 Investigational product/treatment

All subjects will participate in both study arms in a randomized order. Subjects will receive containers with the exact number of capsules for 1 week (14 capsules per week). The daily ingested dose of clenbuterol is 40 μg (2x 20 μg/capsule), which is within the advised daily dose of clenbuterol (40-80 μg/day) for clinical use. The wash-out period in between the study arms will be at least 4 weeks.

4.6 Use of co-intervention (if applicable)

Subjects will be advised to maintain their normal patterns during both supplementation periods with respect to overall daily activities, eating and sleeping.

Regular medication used by the subject will be communicated to the dependent medical doctor and he will advise the researcher whether the subject is allowed to participate in the study or whether the medication interferes with the study outcome parameters (see SPC text of clenbuterol, Document D2). All medication used by the participants will be reported in the electronic case report form (eCRF).

Subjects will be asked to refrain from any extensive physical activities other than daily routines. More specifically, subjects are instructed neither to perform any sport nor to perform walking/cycling longer than 30 minutes, and maximally at low intensity. Subjects will also be asked to perform no heavy ‘household’ tasks three days prior to every visit (e.g. cleaning the windows, gardening or lifting heavy groceries).

4.7 Escape medication (if applicable)

Not Applicable.

5. INVESTIGATIONAL PRODUCT

5.1 Name and description of investigational product(s)

Spiropent - Clenbuterol hydrochloride

Spiropent (Clenbuterol hydrochloride, $C_{12}H_{18}Cl_2N_2O$, **Figure 3**) (See SPC of Spiropent Document D2) is a long-acting selective β_2-adrenergic agonist with sympathomimetic activity which is used for the treatment of obstructive airways diseases with reversible airway narrowing such as bronchial asthma or chronic obstructive bronchitis (19-23). Clenbuterol hydrochloride binds and activates β_2-adrenergic receptors, thereby causing stimulation of adenylyl cyclase, leading to the subsequent synthesis of cyclic-3',5'-adenosine monophosphate (cAMP). Increased levels of cellular cAMP causes smooth muscle cell relaxation (24).

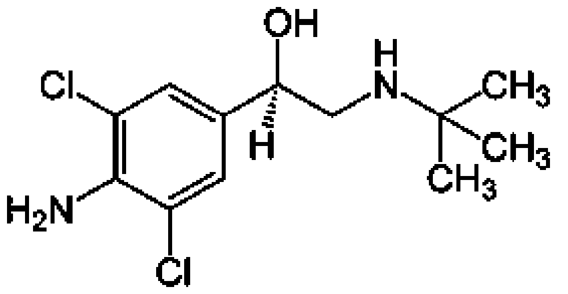

Figure 3. *Chemical structure of clenbuterol*

The normal recommended dose for clinical use of clenbuterol hydrochloride is between 40-80 μg/day (See document D2 for SPC of clenbuterol). After ingestion, clenbuterol hydrochloride is rapidly and fully absorbed in the gastrointestinal region. Maximal plasma concentrations in humans are achieved within 2-3 hours after ingestion, with maximal plasma concentrations of 0.2 ng/mL upon acute 40 μg intake (16). Binding to plasma proteins is 45-68% and clenbuterol is distributed throughout the tissues. Upon prolonged clenbuterol supplementation, plasma clenbuterol levels reach a plateau after 4 days of administration (plateau: 0.5-0.6 ng/mL with 40 μg/day intake) (16). Clenbuterol is metabolised – to a lesser extent – by the liver. In total, 8 metabolites have been found which do not possess any pharmacological activity. The excretion of clenbuterol occurs in two different phases. The T_{50} of the first phase is 1 hour, whereas the T_{50} of the second phase is 34 hours. The main route of excretion is through renal

excretion in an unaltered form (87% within 168 hours post dose) (See SPC clenbuterol, Document D2). Within 168 hours, approximately 97% of the administered dose is excreted.

Each clenbuterol hydrochloride tablet contains 109.98 mg of lactose monohydrate and 0.02 mg of clenbuterol hydrochloride (See SPC of clenbuterol D2). Clenbuterol hydrochloride tablets will be encapsulated by the GMP certified pharmacy of the Radboud UMC (See document D4) to a total dose 20 μg/capsule according to GMP standards. The capsules will be further filled with an inactive compound. Capsules of 20 μg will be ingested twice daily combined with breakfast and dinner.

Placebo

Identical placebo capsules will be created by the pharmacy of Radboud UMC, which contains the same inactive ingredients as the clenbuterol capsules without the active ingredients.

5.2 Summary of findings from non-clinical studies

See Document D2 for a summary of findings from non-clinical studies.

5.3 Summary of findings from clinical studies

See Document D2 for a summary of findings from clinical studies.

5.4 Summary of known and potential risks and benefits

The side effects of clenbuterol are generally known for the pharmacological group of beta-sympathomimetics. These side effects include (see Document D2):

- Cardiac disorders:
    - palpitations, tachycardia, cardiac arrest, in very rare cases atrial fibrillation, in isolated cases, myocardial infarction; of unknown frequency: myocardial ischemia.

- Nervous system disorders:
    - trembling, anxiety, headache, dizziness, insomnia, redness, sweating, excitement.
- Respiratory, breast and mediastinum:
    - paradoxical bronchospasm disorders.
- Disorders of the kidneys and urinary tract:
    - rarely mycosis disorders.
- Disorders of the musculoskeletal system and connective tissue:
    - muscle cramps.
- Metabolism and nutrition disorders:
    - hypokalaemia, hyperglycaemia.
- Disorders of the blood vessels:
    - a decrease or a sharp rise in blood pressure.
- Immune system disorders:
    - skin rash, angioedema.

5.5 Description and justification of route of administration and dosage

The daily recommended dose of clenbuterol hydrochloride is between 40-80 μg/day in adults, which has to be administered orally together with a meal. In this study, we will administer 40 μg of clenbuterol a day (2x 20μg), which is within the daily recommended dose. Capsules have to be administered orally in the morning together with breakfast and in the evening together with dinner.

5.6 Dosages, dosage modifications and method of administration

Clenbuterol tablets will be encapsulated by the pharmacy of Radboud UMC in accordance with GMP and local regulatory guidelines. These capsules are then filled further with an inactive compound. These capsules have a final dose of 20 μg/capsule and can be directly swallowed with a drink (water, fruit juices etc.).

5.7 Preparation and labelling of Investigational Medicinal Product

The study drug, as well as the placebo, will be packaged into bottles containing the correct number of capsules for 1 weeks' supplementation by the pharmacy of Radboud UMC. The

labels will be prepared in accordance with both GMP and local regulatory guidelines, which will thereby fulfil all requirements for labelling (See document D3), including:

- The name, address and telephone number of the researcher (Sten van Beek).
- Pharmaceutical formulation, route of administration, doses
- Batch number
- Information to identify the clinical trial (ABR number and EudraCT).
- Subject number
- Instructions on the use of the product
- Storage conditions
- Expiration date
- Notification to keep out of reach of children

Label texts will be provided in Dutch or English. Since the study will be performed in a double-blinded manner, both clenbuterol containing capsules, as well as the placebo capsules, will be delivered in identical bottles. These bottles will be stored under appropriate conditions at the research facility and kept at room temperature.

5.8 Drug accountability

All study medication will be encapsulated, packed, blinded and labelled by the pharmacy of Radboud UMC. The study medication will thereafter be sent to the hospital pharmacy (AMC) where it will be stored under the appropriate conditions. The researcher (Sten van Beek) will be able to collect the bottles for a subject with a recipe provided by the dependent physician. The bottles will thereafter be stored in temperature-controlled rooms before they are given to a subject. The number of capsules in the bottles will be noted by the researcher in a log before the bottle is given to the subject. The subject is asked to return used bottles at the end of the period and the number of capsules left in the bottles will be counted. Subjects should always return all unused study drugs/empty bottles to the researcher. Left-over medication of subjects who have finished the study will be destroyed by the AMC. All dispensations of bottles will be carefully documented by the researcher in a log and eCRF.

6. NON-INVESTIGATIONAL PRODUCT

6.1 Name and description of non-investigational product(s)

- **Lidocainehydrochloride**:
  - Local anaesthetic (10 mg/mL) used by means of injection. Used during the muscle biopsy.
- **Stable isotope [6,6-2H2] glucose**:
  - The stable isotope [6,6-$^2H_2$] glucose (approved IMPD within several previous studies performed by our research group and approved by the METC: protocol ID 16-3-019, 15-3-046, 15-3-030, 13-3-040, 09-3-039, 09-3-033) will be infused during the clamp to make it possible to measure rates of glucose appearance and disappearance (IMPD of [6,6-$^2H_2$] glucose attached in document D2)
- **Glucose 20% (Baxter)**:
  - 20% w/v, solution for injection. Infused during the hyperinsulinemic-euglycemic clamp
- **Insulin aspart (100 IE/mL) (Novorapid):**
  - Peptide hormone, solution for injection. Infused during the hyperinsulinemic-euglycemic clamp

6.2 Summary of findings from non-clinical studies

Not applicable, all products have been approved for clinical research.

6.3 Summary of findings from clinical studies

Not applicable, all non-investigational products will be used as in standard medical practice:

- SPC lidocaine – see attachment document D2
- SPC Stable isotope [6,6-2H2] glucose – see attachment document D2
- SPC glucose 20% - see attachment document D2
- SPC insulin Aspart – see attachment document D2

6.4 Summary of known and potential risks and benefits

Not applicable, all non-investigational products will be used as in standard medical practice:

- SPC lidocaine – see attachment document D2
- SPC Stable isotope [6,6-2H2] glucose – see attachment document D2
- SPC glucose 20% - see attachment document D2

- SPC insulin Aspart – see attachment document D2

6.5 Description and justification of route of administration and dosage

Not applicable, all non-investigational products will be used as in standard medical practice.

6.6 Dosages, dosage modifications and method of administration

For the muscle biopsy, lidocaine (10 mg/mL) will be injected subcutaneously, as well as under the muscle fascia at the site of the muscle biopsy of the *m.* vastus lateralis.

During the two-step hyperinsulinemic-euglycemic clamp, an insulin infusion (1 IE/mL) will be prepared for intravenous administration according to the following instructions: 0.5 mL of 100 IE/mL Novarapid insulin diluted in 47.5 mL NaCl (9%) and 2 mL blood collected from the participant. The preparation of the insulin infusion will be checked and co-signed by the researcher and a second experienced researcher.

The glucose solution for intravenous administration throughout the hyperinsulinemic-euglycemic clamp will be prepared with a 500 mL glucose bag (20%) to which 64.5 mL [6,6-2H2] glucose and 5 mL KCl (7.5%) will be added. This solution will be infused at variable rates throughout the clamp in order to maintain euglycemia within the subject (~5 mmol/L). Prepared glucose infusion bags will be precisely marked by the researcher. Furthermore, the preparation of the glucose infusion bags will be checked and co-signed by a second experienced researcher.

In addition, during the hyperinsulinemic-euglycemic clamp, a primed (2.4 mg/kg) [6.6-$^2H_2$] glucose isotopic traced will be administered intravenously at a continuous rate of 0.04 mg/kg/min. [6.6-$^2H_2$] glucose isotopic tracer will be added to a 50 mL syringe, which will be marked by the researcher and checked by a second experienced researcher.

6.7 Preparation and labelling of Non Investigational Medicinal Product

All non-investigational products will be stored appropriately. Lidocaine will be transferred to a syringe prior to administration.

6.8 Drug accountability

All non-investigational products have been regularly used for many studies by our research group. All non-investigational products are purchased from the MUMC pharmacy, with the exception of the [6.6-$^{2}H_2$] glucose isotopic tracer which is purchased from the Radboud pharmacy in Nijmegen. All non-investigation products arrive in their original packaging and will be stored as recommended.

7. METHODS

7.1 Study parameters/endpoints

7.1.1 Main study parameter/endpoint

Primary outcome parameter:

- Insulin-stimulated peripheral glucose disposal (Rd) during the high-insulin infusion of the two-step hyperinsulinemic-euglycemic clamp.

7.1.2 Secondary study parameters/endpoints (if applicable)

Secondary study parameters:

- Skeletal muscle GLUT4 translocation

7.1.3 Other exploratory study parameters (if applicable)

Other study parameters:

- Body weight
- Body composition (Bodpod)
- Plasma substrate concentrations
- Heart rate and blood pressure
- Insulin-mediated suppression of hepatic glucose production
- (Sleeping) energy expenditure and substrate oxidation
- Skeletal muscle glycogen and lipid content
- Gene and protein expression in skeletal muscle

7.2 Randomisation, blinding and treatment allocation

Participants that come to the MRUM for an initial screening will be given an code. In case the subject is allowed to participate in the study, a randomization will be performed to allocate the subject in either group A or B. We will use randomization to ensure that the order in which the medication is given (i.e. clenbuterol – placebo and placebo – clenbuterol) occurs equally frequent in the entire subject group. The randomization will be performed by an independent researcher and will be performed with the help of the website www.randomizer.org, and will be performed in groups of 4 participants for the entire study. This randomization list containing the participant codes and allocation to the treatment A or B will be send to the pharmacy of

Radboud UMC. Accordingly, the pharmacy of Radboud UMC prepares the study bottles for the participants. Four envelopes containing the unblinding key for the intervention arms A and B will be prepared by the pharmacy of Radboud UMC. These envelopes will be send to the project leader (Dr. J. Hoeks), the independent physician (Dr. R. Henry), the secretary of the Department of Nutrition and Movement Sciences (Yolanda Verhaegen) and the responsible medical physician of the study (Dr. B. Havekes). All these players are responsible to keep these envelopes safe. The envelopes will be opened only in situations where de-blinding of the study is necessary. In view of the nature of this study no indications for breaking the randomization code are predetermined, neither expected. In a remote possibility of any untoward effect, the persons mentioned above can break the blinding code for the particular participant.

7.3 Study procedures

8.3.1. Recruitment

Subjects will be recruited in Maastricht and surroundings by means of posters and advertisements in local newspapers and online (See document E3). Also, subjects that previously participated in other studies at the Department of Nutrition and Movement Sciences at Maastricht University or were not eligible to participate in other studies will be approached by means of a standardized email providing a short explanation of the study design (See Document K6). This type of recruitment will only occur if subjects indicated that they want to be approached for participation in future studies (signed consent) and appear to fulfill the inclusion criteria. The researchers will contact individuals who are interested in participating in the study by telephone, only after the individual has sought contact with the researcher on his or her accord first. By contacting responders by telephone first, the burden of travel and time effort is reduced for the potential subject and researchers. In the telephone interview, the goal of the study will be explained, and the basic inclusion criteria will be discussed. When responders are interested, they will receive detailed subject information via e-mail or mail accompanied by a general brochure (provided by the Dutch government) about participating in a medical study. They will be instructed to read this information carefully and to ask questions if things are unclear. The researcher will contact the possible participant again at least 7 days after the study information was received by the participant. If responders want to participate after reading the study information and seem to be eligible, they are invited for a screening.

8.3.2. Screening

Only when the informed consent is signed, participants can undergo the screening. Both the participant as well as the researcher will sign the informed consent before the start of the screening. The screening will include the following procedures:

- together with the participant, a questionnaire will be filled on health status, physical activity and medical history (see document F1)
- a fasting blood sample will be drawn to determine clinical health parameters including parameters for liver and kidney function
- physical examination, like body weight and length
- An ECG will be taken
- blood pressure will be measured 3 times in row at the contra-dominant arm

A total of 15 ml blood will be drawn.

Body weight

During the screening, body weight will be measured by means of a digital balance with an accuracy of 0.001 kg (seca, seca GmbH & Co, Hamburg, Germany).

Medical history and physical activity questionnaires

Questionnaires regarding medical history and physical activity will be filled in by the subjects during the screening (See document F1).

Blood samples and invasive biomarkers

Fasted blood samples will be taken during the screening by means of venepuncture. Blood samples will be used for the determination of clinical health parameters. The analyses will be performed by the Maastricht University Medical Centre.

8.3.3. Measurements during study period

Body weight

During the supplementation periods (T = 1, 8 and 15 days of each study arm), body weight will be measured by means of a digital balance with an accuracy of 0.001 kg (seca, seca GmbH & Co, Hamburg, Germany).

Heart rate and blood pressure

Resting heart rate and blood pressure will be measured 3 times in a row in the contra-dominant arm at three occasions of each study arm (T = 1, 8 and 15 days/study arm) by means of an automatic inflatable cuff (Omron Healthcare, Hamburg, Germany).

Indirect calorimetry

Energy expenditure and substrate oxidation will be measured by means of an open circuit respirometry with an automated ventilated hood system (Omnical, Maastricht Instruments, Maastricht University, Maastricht, The Netherlands). This will be performed both acutely (during the first 4h after initial intake of the first capsule) and after 2 weeks of supplementation.

Muscle biopsies

Muscle biopsies will be taken from all subjects after both acute (4h after first supplement) and long-term (2-weeks) clenbuterol/placebo supplementation. Muscle biopsies from the m. vastus lateralis will be taken and performed under local anaesthesia with Lidocaine 1.0% solution (10mg/mL) without adrenalin. A side-cutting needle will be used to acquire the muscle tissue, following the Bergström method (25). The leg of the biopsy will be randomized to exclude influence of a relatively more trained muscle versus a relatively less trained muscle. The biopsy material will be processed *ex vivo* immediately and will be frozen in isopentane cooled with liquid nitrogen and stored at minus 80 °C for later analyses. For these analyses, about 300mg of muscle tissue is needed, being a standard amount of tissue that is to be acquired in each biopsy.

Femoral artery flow mediated dilation (FMD)

Femoral artery flow mediated dilation (FMD) is assessed by Echo-Doppler (MyLab^{TM}Gamma, Esaote) by using a 7.5-MHz transducer and recording of echo images on the laptop. After a 3-minute reference period, the pneumatic cuff placed around the participant’s leg is inflated 50 mmHg above systolic pressure for 5 minutes, causing distal hypoxia. Upon cuff-release

reactive hyperemia ensues. The echo images are processed automatically to determine the diameter profiles over the entire 15 min of the femoral artery FMD measurement using a custom-written Matlab program (MyFMD, Prof. A.P.G. Hoeks, Department of Biomedical Engineering, Maastricht University, Maastricht, the Netherlands). The FMD response is then quantified as the maximal percentage change in post occlusion arterial diameter relative to baseline diameter.

Bodpod

The Bod Pod® (Cosmed) will be used to determine body composition and measures fat mass, fat free mass, total body mass and estimates resting metabolic rate (RMR) via whole body densitometry. The Bod Pod utilizes the displacement of air during the measurement to determine body composition. The Bod Pod technology is fundamentally the same as the underwater (hydrostatic) weighing but uses air instead of water. The volume of air a person's body displaces is measured while sitting inside a comfortable chamber for two 50- second measurements. It is a safe, non-invasive, easy-to-use and quick tool for measuring body composition. The duration of the measurement is approximately 5 minutes.

Metabolic chamber: sleeping energy expenditure and substrate oxidation

The night from day 14 to 15 of each study arm will be spend by subjects in a respiration chamber, which is equipped with indirect calorimetry (Omnical, Maastricht Instruments, Maastricht University, Maastricht, The Netherlands) and measures oxygen consumption and carbon dioxide production. Based on these values, the sleeping metabolic rate and substrate oxidation will be calculated. Subjects will receive a standardized meal before they enter the chamber and will enter at around 18:00 hours. The respiration chamber is a 14m^2 room furnished with a bed, chair, desk, TV, telephone, computer, washbowl and toilet. The room is ventilated with fresh air. Privacy is warranted, as the subject in the chamber can close the curtains of the outside windows.

Two-step hyperinsulinemic-euglycemic clamp

A two-step hyperinsulinemic-euglycemic clamp will be performed as described by DeFronzo et al. (26) in the post-absorptive state beginning after a 12-hour overnight fast the morning after the chambers. A teflon cannula will be inserted into antecubital veins of one arm for the infusion of glucose tracer, insulin and glucose. Another cannula will be inserted retrogradely

into a superficial dorsal hand vein. This venous blood will be arterialized by placing the hand into a hotbox, which blows warm air (50°C).

After taking fasting blood samples, a primed continuous infusion of [6,6 $^2H_2$] glucose (priming dose: 2.4 mg/kg; continuous infusion: 0.04 mg•kg$^-$1•min^{-1}) will be administered (t =0 min). This is a naturally occurring isotope, which is in no way harmful to humans and will be obtained from the pharmacy of the Academic Hospital Nijmegen. At t= 120 min a muscle biopsy will be taken from the *m. vastus lateralis*. After 150 minutes of isotopic equilibration, four blood samples will be obtained at 10-min intervals (t=150, 160, 170 and 180 min) for the determination of basal blood substrates, whole body glucose disposal and hepatic glucose production. Indirect calorimetry (ventilated hood) will be performed during this last half hour of the baseline equilibration period to determine substrate oxidation (t =150 - 180).

At t =180 minutes, a 3-h low primed constant infusion of insulin is started (10 mU/m^2/min, Novorapid, Novo Nordisk) to asses hepatic insulin resistance. Plasma glucose levels will be clamped at ~5 mmol/L by variable co-infusion of 20% glucose. At regular time points (every 5 to 10 minutes), a small volume of blood (0.9 ml) will be sampled for immediate determination of plasma glucose concentration. When necessary, glucose infusion rate will be adjusted to obtain plasma glucose levels of ~5.0 mmol/L (euglycemia). Indirect calorimetry will be performed at t =330-360 min. During this time period, four blood samples will be obtained at 10-min intervals for the determination of whole-body glucose disposal and hepatic glucose production (t =330, 340, 350 and 360). At t=360 the 2,5-h high primed constant insulin infusion is started (40 mU/m^2/min), in order to fully stop the hepatic glucose production and only study the rate of disappearance (Rd) as a measure for skeletal muscle insulin sensitivity. Here again plasma glucose levels will be clamped at ~5 mmol/L by variable co-infusion of 20% glucose. At regular time points (every 5 to 10 minutes), a small volume of blood (0.9 ml) will be sampled for immediate determination of plasma glucose concentration. When necessary, glucose infusion rate will be adjusted to obtain plasma glucose levels of ~5 mmol/L (euglycemia). Indirect calorimetry will be performed at t=480-510 min. During this time period, four blood samples will again be obtained at 10-min intervals (t =480, 490, 500 and 510). During the indirect calorimetry during the clamp, respiratory gas exchange will be measured using an open-air circuit respirometry with an automated ventilated hood system (Omnical, Maastricht Instruments, Maastricht University, Maastricht, The Netherlands).

8.3.4. Analyses

Blood samples

Fasting blood samples acquired during the screening will be analyzed by the Maastricht University Medical Centre for determination of clinical health parameters.

Collection of fasting blood samples will be performed in the appropriate tubes on day 1, 8 and 15 of the supplementation periods. Blood samples will be centrifuged at high-speed and blood plasma will be transferred to a new tube. Plasma will be immediately flash-frozen in liquid nitrogen and stored in -80 degrees Celsius until further analyses.

Glycogen content

Muscle glycogen content will be analysed in the frozen muscle biopsies by means of a commercial glycogen kit (Abcam).

Gene and protein expression

Skeletal muscle biopsies will be assessed for gene and protein expression of several metabolic pathways (e.g. insulin signalling pathway, AMPK-pathway, mTORC2 pathway and mitochondrial markers) by means of RT-qPCR or Western blot, respectively.

Immunohistochemistry

Determination of skeletal muscle GLUT4-translocation and lipid content will be performed by means of immunohistochemistry and assessed by wide-field microscopy.

7.4 Withdrawal of individual subjects

Subjects can leave the study at any time for any reason if they wish to do so without any consequences. The investigator can decide to withdraw a subject from the study for urgent medical reasons.

7.4.1 Specific criteria for withdrawal (if applicable)

The investigator can decide to withdraw a participant from the study for the following reasons:

- Non-medical reasons e.g. request by the participant or non-compliance to the study
- Medical reasons argued to be significant by the medical responsible doctor/researcher and/or participant.

- Protocol violation
- In case of illness or changes use of medication of the participant

In all cases, the researcher together with the medical responsible doctor decides whether the participant may continue the study or not.

7.5 Replacement of individual subjects after withdrawal

The subject will not be replaced upon withdrawal from the study since subject drop-out has been anticipated for in the sample size calculation.

7.6 Follow-up of subjects withdrawn from treatment

No follow-up of the subject will be performed upon withdrawal. In case a subject drops out of the study due to medical complication, the research team will provide the subject with guidance to the responsible medical doctor of the study and eventually be referred to the general practitioner.

7.7 Premature termination of the study

In case of premature termination of the study, the METC AzM/UM and CCMO (competent authority) will be informed within 15 days after termination. Both the METC and CCMO will be notified regarding the reason of premature termination of the study using the appropriate documents.

8. SAFETY REPORTING

9.1 Temporary halt for reasons of subject safety

In accordance to section 10, subsection 4, of the WMO, the sponsor will suspend the study if there is sufficient ground that continuation of the study will jeopardise subject health or safety. The sponsor will notify the accredited METC without undue delay of a temporary halt including the reason for such an action. The study will be suspended pending a further positive decision by the accredited METC. The investigator will take care that all subjects are kept informed.

9.2 AEs, SAEs and SUSARs

9.2.1. Adverse events (AEs)

Adverse events are defined as any undesirable experience occurring to a subject during the study, whether or not considered related to the investigational product . All adverse events reported spontaneously by the subject or observed by the investigator or his staff will be recorded.

9.2.2. Serious adverse events (SAEs)

A serious adverse event is any untoward medical occurrence or effect that

- results in death;
- is life threatening (at the time of the event);
- requires hospitalisation or prolongation of existing inpatients' hospitalisation;
- results in persistent or significant disability or incapacity;
- is a congenital anomaly or birth defect; or
- any other important medical event that did not result in any of the outcomes listed above due to medical or surgical intervention but could have been based upon appropriate judgement by the investigator.

An elective hospital admission will not be considered as a serious adverse event.

The investigator will report all SAEs to the sponsor without undue delay after obtaining knowledge of the events. The sponsor will report the SAEs through the web portal *ToetsingOnline* to the accredited METC that approved the protocol, within 7 days of first knowledge for SAEs that result in death or are life threatening followed by a period of maximum of 8 days to complete the initial preliminary report. All other SAEs will be

reported within a period of maximum 15 days after the sponsor has first knowledge of the serious adverse events.

9.2.3. Suspected unexpected serious adverse reactions (SUSARs)

Adverse reactions are all untoward and unintended responses to an investigational product related to any dose administered.

Unexpected adverse reactions are SUSARs if the following three conditions are met:

1. the event must be serious (see chapter 9.2.2);
2. there must be a certain degree of probability that the event is a harmful and an undesirable reaction to the medicinal product under investigation, regardless of the administered dose;
3. the adverse reaction must be unexpected, that is to say, the nature and severity of the adverse reaction are not in agreement with the product information as recorded in:
    - Summary of Product Characteristics (SPC) for an authorised medicinal product;
    - Investigator’s Brochure for an unauthorised medicinal product.

The sponsor will report expedited the following SUSARs through the web portal *ToetsingOnline* to the METC:

- SUSARs that have arisen in the clinical trial that was assessed by the METC;
- SUSARs that have arisen in other clinical trials of the same sponsor and with the same medicinal product, and that could have consequences for the safety of the subjects involved in the clinical trial that was assessed by the METC.

The remaining SUSARs are recorded in an overview list (line-listing) that will be submitted once every half year to the METC. This line-listing provides an overview of all SUSARs from the study medicine, accompanied by a brief report highlighting the main points of concern.

The expedited reporting of SUSARs through the web portal Eudravigilance or ToetsingOnline is sufficient as notification to the competent authority.

The sponsor will report expedited all SUSARs to the competent authorities in other Member States, according to the requirements of the Member States.

The expedited reporting will occur not later than 15 days after the sponsor has first knowledge of the adverse reactions. For fatal or life threatening cases the term will be maximal 7 days for a preliminary report with another 8 days for completion of the report.

9.3. Annual safety report

In addition to the expedited reporting of SUSARs, the sponsor will submit, once a year throughout the clinical trial, a safety report to the accredited METC, competent authority, and competent authorities of the concerned Member States.

This safety report consists of:

- a list of all suspected (unexpected or expected) serious adverse reactions, along with an aggregated summary table of all reported serious adverse reactions, ordered by organ system, per study;
- a report concerning the safety of the subjects, consisting of a complete safety analysis and an evaluation of the balance between the efficacy and the harmfulness of the medicine under investigation.

9.4. Follow-up of adverse events

All AEs will be followed until they have abated, or until a stable situation has been reached. Depending on the event, follow up may require additional tests or medical procedures as indicated, and/or referral to the general physician or a medical specialist.

SAEs need to be reported till end of study within the Netherlands, as defined in the protocol

9.5. [Data Safety Monitoring Board (DSMB) / Safety Committee]

Not applicable.

10. STATISTICAL ANALYSIS

Statistical analyses will be performed on both the subject population that was randomized into the study (intention-to-treat) and on the subjects that completed the study (per protocol). The first analysis prevents attrition bias. However, since the main aim of this study is to analyse the effects of clenbuterol vs. placebo supplementation on skeletal muscle glucose uptake, we will also perform a per protocol analysis. Comparing the intention-to-treat analysis with the per-protocol analysis will give us valuable information regarding the amount of bias introduced.

Statistical analyses will be performed using SPSS for Mac iOS (IBM, version 23). All data will be tested for normality by visual inspection of data histograms and by means of Kolmogorov-Smirnov normality tests. When normally distributed, all data will be analysed with a paired Students T-test to analyse statistical differences between clenbuterol vs. placebo groups. In case of non-normally distributed data, a non-parametrical Wilcoxon signed-ranked test will be performed. Differences will be considered statistically significant when tested two-sided $p < 0.05$.

Data will be presented as mean ± standard error of the mean in case of normally distributed data. In case of non-normally distributed data, data will be presented as median and 25% or 75%. The hypothesis being tested during this study is:

H_0: $\mu_{placebo} = \mu_{clenbuterol}$

H_A: $\mu_{placebo} \neq \mu_{clenbuterol}$

During the study, only data from subjects who completed both study periods will be included in the data analysis. Missing values will not be replaced and, if a data for a specific parameter is missing, the subject in question will not contribute to the analysis of said parameter.

10.2. Primary study parameter(s)

The primary outcome parameter of this study is insulin-stimulated peripheral glucose disposal rate (Rd) (expressed as μmol/kg/min) which will be obtained during the high-insulin infusion

rate step during the two-step hyperinsulinemic-euglycemic clamp. This parameter is a numerical variable and will be expressed as mean ± standard error, minimum and maximum. Statistical analyses of glucose disposal rate will be performed by means of a paired students' T-test or Wilcoxon signed-ranked test based on the normality.

10.3. Secondary study parameter(s)

The secondary parameter is a numerical variable and will be presented as mean ± standard error, minimum and maximum or median, based on the normality of the data. The secondary parameter will be analysed by means of a Students' T-test of Wilcoxon signed-rank test. Correlations between primary and secondary parameters will be performed with a Pearson's R correlation for normally distributed data. A Spearman's correlation test will be applied for non-normally distributed data.

10.4. Other study parameters

Subject characteristics will not be statistically analysed and will merely be presented as minimum, maximum, mean and standard error of the mean. Results acquired at the beginning, half-way and end of a supplementation period will be compared by means of a two-way ANOVA with a Bonferonni post-hoc test. This is performed to statistically analyse effects of clenbuterol/placebo supplementation on different variables upon acute and prolonged supplementation.

10.5. Interim analysis (if applicable)

Not applicable.

11. ETHICAL CONSIDERATIONS

11.2. Regulation statement

This study will be conducted according to the principles of the Declaration of Helsinki (64th WMA General Assembly, Fortaleza, Brazil, October 2013) and in accordance with the Medical Research Involving Human Subjects Act (WMO). The study will be conducted in compliance with International Conference on Harmonization Good Clinical Practice. The study will be approved by local medical ethics committee and local authorities before start of the study.

11.3. Recruitment and consent

Subjects will be recruited in Maastricht and surroundings by means of posters and advertisements in local newspapers and online (See document E3). Also, subjects that previously participated in other studies at the Department of Nutrition and Movement Sciences at Maastricht University or were not eligible to participate in other studies will be approached by means of a standardized email providing a short explanation of the study design (See Document K6). This type of recruitment will only occur if subjects indicated that they want to be approached for participation in future studies (signed consent) and appear to fulfill the inclusion criteria. The researchers will contact individuals who are interested in participating in the study by telephone, only after the individual has sought contact with the researcher on his or her accord first. By contacting responders by telephone first, the burden of travel and time effort is reduced for the potential subject and researchers. In the telephone interview, the goal of the study will be explained, and the basic inclusion criteria will be discussed. When responders are interested, they will receive detailed subject information via e-mail or mail accompanied by a general brochure (provided by the Dutch government) about participating in a medical study. They will be instructed to read this information carefully and to ask questions if things are unclear. The researcher will contact the possible participant again at least 7 days after the study information was received by the participant. If responders want to participate after reading the study information and seem to be eligible, they are invited for a screening.

11.4. Objection by minors or incapacitated subjects (if applicable)

Not applicable.

11.5. Benefits and risks assessment, group relatedness

Participation in this study will not result in any health benefits. During this study, the effect of clenbuterol vs. placebo will be investigated within the same subjects due to the cross-over design. The main benefit of this study is the acquired knowledge regarding the role of beta-2 adrenergic receptors in skeletal muscle glucose uptake and the glucose homeostasis in general. Furthermore, this study could stimulate the development of super biased beta-2 adrenergic ligands by pharmaceutical companies to prevent and treat T2DM.

Participation to this study will pose an intermediate risk to subject's health. The main burdens for the subjects are: time spend to participate in the study, potential adverse effects of clenbuterol, invasive sample collection, non-invasive measurements and unexpected medical findings. These burdens will be discussed into more detailed below.

Time spend to participate in the study

During this study, subjects will visit the University of Maastricht at 6 different occasions (excl. screening) in a period of 2 months. The total time which will be spend to the study is approximately 67 hours, which is excluding travelling time. To minimize this burden, the visits to the University will be carefully planned together with the subject.

Potential adverse effects of clenbuterol

Clenbuterol could potentially induce side effects, including headache, dizziness, heartburn, tremor, muscle ache and spasms. For a full list of all side effects, please see the SPC of clenbuterol in Document D2.

Invasive sample collection

During this study, several invasive sample collections will be performed, including blood sample collection, muscle biopsies and the hyperinsulinemic-eugluycemic clamp. These measurements could be associated with local hematoma or bruise development. However, due to the state-of-the-art techniques, risks for infection or prolonged bleeding will be minimized. To further minimize these risks, pressure bandage will be placed which will have to be worn by the subject for at least 24 hours and subjects are recommended to refrain from

any intensive exercise or heavy lifting. Within a couple of days, bruises will disappear. Finally, the muscle tissue collection will be performed by an experienced medical doctor.

Non-invasive measurements

Blood pressure and heart rate measurements will be performed by means of an automatic inflatable cuff. These measurements might feel a bit uncomfortable, but do not induce detrimental health effects.

Unexpected medical findings

During the screening and measurements of the study, unexpected medical findings might be found. Subjects will always be informed regarding unexpected findings and this information will also be communicated to the general physician. If a subject does not want to be informed about unexpected medical findings, the subject is not allowed to participate.

Risk-benefit assessment

The current study could potentially contribute to the development of a novel class of medication to prevent and treat T2DM. Since the prevalence of T2DM is reaching epidemic levels, we believe that the risks and time investment of this study our outweighed by the benefits.

11.6. Compensation for injury

The sponsor/investigator has a liability insurance which is in accordance with article 7 of the WMO. The sponsor also has an insurance which is in accordance with the legal requirements in the Netherlands (Article 7 WMO). This insurance provides cover for damage to research subjects through injury or death caused by the study.

The insurance applies to the damage that becomes apparent during the study or within 4 years after the end of the study.

1. € 650.000,-- (i.e. six hundred and fifty thousand Euro) for death or injury for each subject who participates in the Research;

2. € 5.000.000,-- (i.e. five million Euro) for death or injury for all subjects who participate in the Research;
3. € 7.500.000,-- (i.e. seven million and five hundred thousand Euro) for the total damage incurred by the organisation for all damage disclosed by scientific research for the Sponsor as 'verrichter' in the meaning of said Act in each year of insurance coverage.

11.7. Incentives (if applicable)

Subjects will receive a financial compensation of €500 for completing the study (See Attachment J1). In case of premature termination of the study, a reduced fee will be transferred which is dependent on the time investment and invasive procedures performed. The screening will not be compensated for since this could provide beneficial information for the participant. Compensation of travel costs will be made for all subjects participating in the study. This compensation will be maximally 19 eurocent per kilometre when travelling by car and complete cover of expenses when travelling by public transport.

12. ADMINISTRATIVE ASPECTS, MONITORING AND PUBLICATION

12.2. Handling and storage of data and documents

All information which will be obtained from the subject prior to and during the study will be kept private and protected. Prior to the start of the screening, subjects will be given an code (clen-followed by a number) which will not be changed during the study. The code is linked with the name, address, date of birth and telephone number of the subject in a password protected file. This file can only be accessed by the research team (Drs. Sten van Beek and Dr. Joris Hoeks), with exception of technicians. The subject code will be used for all purposes for subject identification during the study.

The analyses of documents, data or samples outside the scope of this project will always require the permission of the subject. However, when collected data and samples obtained during the study can be applied during future studies within the scope of the study, no permission will be asked.

Subjects will be given the opportunity to obtain information regarding their personal study results either verbally or in writing. This will always be performed in accordance to the privacy statement (Algemene Verordening Gegevensbescherming, AVG). In case of chance findings obtained during the screening (including elevated blood pressure or abnormal blood plasma values), these events will be noted in the CRF of the subject. In collaboration with the dependent physician, suitable action will be taken.

All samples collected during this study will be stored for 15 years. Storage of muscle biopsies will be performed in the -80 degrees freezers at the Department of Nutrition and Movement Sciences at Maastricht university, whereas blood samples will be stored at the BioBank Maastricht University Medical Centre+. Approval will be asked during the informed consent and if subject decline the storage of the material, they will not be allowed to participate in the study. After 15 years, all the study material will be destroyed. Until then, only the research team, IGJ, METC and monitors have access to the research data and documents.

12.3. Monitoring and Quality Assurance

Not applicable.

12.4. Amendments

A ‘substantial amendment’ is defined as an amendment to the terms of the METC application, or to the protocol or any other supporting documentation, that is likely to affect to a significant degree:

- the safety or physical or mental integrity of the subjects of the trial;
- the scientific value of the trial;
- the conduct or management of the trial; or
- the quality or safety of any intervention used in the trial.

All substantial amendments will be notified to the METC and to the competent authority.

Non-substantial amendments will not be notified to the accredited METC and the competent authority, but will be recorded and filed by the sponsor.

12.5. Annual progress report

The sponsor/investigator will submit a summary of the progress of the trial to the accredited METC once a year. Information will be provided on the date of inclusion of the first subject, numbers of subjects included and numbers of subjects that have completed the trial, serious adverse events/ serious adverse reactions, other problems, and amendments.

12.6. Temporary halt and (prematurely) end of study report

The sponsor will notify the accredited METC and the competent authority of the end of the study within a period of 90 days. The end of the study is defined as the last patient’s last visit.

The sponsor will notify the METC immediately of a temporary halt of the study, including the reason of such an action

In case the study is ended prematurely, the sponsor will notify the accredited METC and the competent authority within 15 days, including the reasons for the premature termination.

Within one year after the end of the study, the investigator/sponsor will submit a final study report with the results of the study, including any publications/abstracts of the study, to the accredited METC and the Competent Authority.

12.7. Public disclosure and publication policy

Both positive and negative results of this study will be published in peer-reviewed scientific journals. The publication policy is in agreement with the CCMO publication statement.

13. STRUCTURED RISK ANALYSIS

13.2. Potential issues of concern

a. Level of knowledge about mechanism of action

It is currently unknown whether supplementation with a selective β_2-agonist enhances skeletal muscle glucose uptake through the mTORC2 pathway in humans. However, we have compelling evidence from both *in vitro* and *in vivo* models that supplementation with a β_2-agonist beneficially affects skeletal muscle glucose uptake, and glucose homeostasis, through the activation of the mTORC2 pathway (See Chapter 1 Introduction), thereby strengthening our working hypothesis.

b. Previous exposure of human beings with the test product(s) and/or products with a similar biological mechanism

Clenbuterol hydrochloride is used as an asthma medication in several European countries and effects have been reported in several peer-reviewed articles. Furthermore, extensive research has been performed on the safety and pharmacokinetics of the medicine (See Document D2). These studies have all been performed on patients with obstructive airway diseases.

c. Can the primary or secondary mechanism be induced in animals and/or in *ex-vivo* human cell material?

Multiple studies have shown an increase in *in vitro* skeletal muscle glucose uptake upon β_2-agonist treatment (27-30), although the precise underlying molecular mechanisms remained unclear. Recently, it has been shown that incubation with clenbuterol significantly enhanced glucose uptake in both cultured L6 muscle cells and human primary myotubes through the activation of mTORC2 (9). In addition, clenbuterol supplementation (30 mg/L in the drinking water) significantly improved glucose tolerance in both high-fat diet-induced obese mice and Goto-Kakizaki rats, two-well established animal models for diabetes (9). In addition, these beneficial effects were abolished in β_1/β_2-knockout mice, indicating the physiological relevance of this pathway (9). These effects were also observed upon a low-dose of clenbuterol supplementation (See Chapter 1 introduction).

d. Selectivity of the mechanism to target tissue in animals and/or human beings

The beneficial effects seen in rodents are most likely mediated by the skeletal muscle, which has a high abundancy of the β_2-adrenergic receptor (9-11). In addition, these effects might partially be mediated by the liver, as the liver also contains high amounts of the β_2-adrenergic receptor (31, 32). These effects will also be investigated during the study by means of the low-insulin infusion step during the two-step hyperinsulinemic-euglycemic clamp (i.e. insulin-mediated suppression of hepatic glucose output). For further information regarding the toxicology, please see the SPC of clenbuterol (Document D2).

e. Analysis of potential effect

The daily recommended dose of clenbuterol is between 40 and 80 μg/day. The dose used during this study falls within the daily recommended dose and, thereby, does not induce toxic doses. The effect of clenbuterol on skeletal muscle glucose uptake is known, in mice, to be dose-dependent (*manuscript in preparation).* Common side effects do not pose a hazard for overall health. For further detailed information regarding side effects, please see SPC text of clenbuterol.

f. Pharmacokinetic considerations

Clenbuterol hydrochloride is rapidly and fully absorbed after oral administration in the gastrointestinal region. Maximal plasma concentrations are achieved within 2-3 hours after ingestion (with maximal concentrations of 0.2 ng/mL with an acute 40 μg intake) and is distributed evenly throughout the tissues. Binding to plasma proteins is 45-68%. Upon prolonged clenbuterol supplementation, plasma levels reach a plateau within 4 days after initial administration (plateau: 0.5-0.6 ng/mL with 40 μg/day) (REF: Yamamoto).

The metabolism of clenbuterol is performed, to a lesser extent, by the liver. In total, 8 metabolites have been found which do not have any pharmacological activity. The excretion of clenbuterol occurs in two phases. The T_{50} of the first phase is 1 hour, whereas the second phase is 34 hours. The main route of excretion is through renal excretion in an unaltered form (87% within 168 hours post dose). Within 168 hours, approximately 97% of the administered dose is excreted.

g. Study population

The study population will consist of healthy, lean (BMI: 20-25 kg/m^2) male subjects aged between 18-30 years old.

h. Interaction with other products

Please see SPC text of Clenbuterol (Document D2).

i. Predictability of effect

Please see SPC text of Clenbuterol (Document D2).

j. Can effects be managed?

Any side effects that emerge with clenbuterol supplementation will disappear upon withdrawal. Participants will receive a card with the relevant information of the study that a physician needs to know in case of an emergency (See Document F3). Furthermore, the physician can, in case of emergency, contact the independent physician (Dr. R. Henry), dependent medical physician (Dr. B. Havekes), the secretary of the department of Nutrition and Movement Sciences (Yolanda Verhaegen) or the principle investigator (Dr. J. Hoeks) for the unblinding key to decipher whether the subject has clenbuterol or placebo supplementation. In less emergent situations, the subject can contact the researcher (Sten van Beek) or the principle investigator (Dr J. Hoeks) during working hours. In that case, the responsible medical doctor will be contacted to decide whether the subject should be excluded from the study.

13.3. Synthesis

The risks of the measurements during this study are low which is highlighted by the low prevalence of adverse effects. This is mainly due to the state-of-the-art techniques that are applied and the sterile working methods. In addition, the burden of time investment is relatively low (~67 hours spread over 2 months including two overnight stays). A burden of this study is

the adverse effects that can be caused by the clenbuterol supplement. However, within the clinical range, these adverse effects are minimal and these effects will disappear upon discontinuation of the supplement. To further reduce the risk of adverse effects, we deliberately choose for a standard dose, healthy subjects and a cross-over design. We believe that these risks are acceptable for the subjects since this study can potentially open fundamentally new therapeutic methods to treat insulin resistance in T2DM.

14. Supplementary References

1. World Health Organization. Obesity and Overweight Fact Sheet 2016 [updated June. Available from: http://www.who.int/mediacentre/factsheets/fs311/en/.
2. World Health Organization. Diabetes Fact Sheet 2017 [updated July Available from: http://www.who.int/mediacentre/factsheets/fs312/en/.
3. van der Lans AA, Hoeks J, Brans B, Vijgen GH, Visser MG, Vosselman MJ, et al. Cold acclimation recruits human brown fat and increases nonshivering thermogenesis. J Clin Invest. 2013;123(8):3395-403.
4. van Marken Lichtenbelt WD, Vanhommerig JW, Smulders NM, Drossaerts JM, Kemerink GJ, Bouvy ND, et al. Cold-activated brown adipose tissue in healthy men. N Engl J Med. 2009;360(15):1500-8.
5. Hanssen MJ, Hoeks J, Brans B, van der Lans AA, Schaart G, van den Driessche JJ, et al. Short-term cold acclimation improves insulin sensitivity in patients with type 2 diabetes mellitus. Nat Med. 2015;21(8):863-5.
6. Meex RC, Schrauwen-Hinderling VB, Moonen-Kornips E, Schaart G, Mensink M, Phielix E, et al. Restoration of muscle mitochondrial function and metabolic flexibility in type 2 diabetes by exercise training is paralleled by increased myocellular fat storage and improved insulin sensitivity. Diabetes. 2010;59(3):572-9.
7. Colberg SR, Sigal RJ, Fernhall B, Regensteiner JG, Blissmer BJ, Rubin RR, et al. Exercise and type 2 diabetes: the American College of Sports Medicine and the American Diabetes Association: joint position statement executive summary. Diabetes Care. 2010;33(12):2692-6.
8. O'Neill HM. AMPK and Exercise: Glucose Uptake and Insulin Sensitivity. Diabetes Metab J. 2013;37(1):1-21.
9. Sato M, Dehvari N, Oberg AI, Dallner OS, Sandstrom AL, Olsen JM, et al. Improving type 2 diabetes through a distinct adrenergic signaling pathway involving mTORC2 that mediates glucose uptake in skeletal muscle. Diabetes. 2014;63(12):4115-29.
10. Liggett SB, Shah SD, Cryer PE. Characterization of beta-adrenergic receptors of human skeletal muscle obtained by needle biopsy. Am J Physiol. 1988;254(6 Pt 1):E795-8.
11. Sillence MN, Matthews ML. Classical and atypical binding sites for beta-adrenoceptor ligands and activation of adenylyl cyclase in bovine skeletal muscle and adipose tissue membranes. Br J Pharmacol. 1994;111(3):866-72.
12. Cypess AM, Chen YC, Sze C, Wang K, English J, Chan O, et al. Cold but not sympathomimetics activates human brown adipose tissue in vivo. Proc Natl Acad Sci U S A. 2012;109(25):10001-5.
13. Castle A, Yaspelkis BB, 3rd, Kuo CH, Ivy JL. Attenuation of insulin resistance by chronic beta2-adrenergic agonist treatment possible muscle specific contributions. Life Sci. 2001;69(5):599-611.
14. Pan SJ, Hancock J, Ding Z, Fogt D, Lee M, Ivy JL. Effects of clenbuterol on insulin resistance in conscious obese Zucker rats. Am J Physiol Endocrinol Metab. 2001;280(4):E554-61.

15. George I, Xydas S, Mancini DM, Lamanca J, DiTullio M, Marboe CC, et al. Effect of clenbuterol on cardiac and skeletal muscle function during left ventricular assist device support. J Heart Lung Transplant. 2006;25(9):1084-90.
16. Yamamoto I, Iwata K, Nakashima M. Pharmacokinetics of plasma and urine clenbuterol in man, rat, and rabbit. J Pharmacobiodyn. 1985;8(5):385-91.
17. Kamalakkannan G, Petrilli CM, George I, LaManca J, McLaughlin BT, Shane E, et al. Clenbuterol increases lean muscle mass but not endurance in patients with chronic heart failure. J Heart Lung Transplant. 2008;27(4):457-61.
18. Hoeks J, van Herpen NA, Mensink M, Moonen-Kornips E, van Beurden D, Hesselink MK, et al. Prolonged fasting identifies skeletal muscle mitochondrial dysfunction as consequence rather than cause of human insulin resistance. Diabetes. 2010;59(9):2117-25.
19. Su WJ, Perng RP. Spiropent (clenbuterol): another choice for patients with chronic reversible airways obstruction. Zhonghua Yi Xue Za Zhi (Taipei). 1991;47(1):13-7.
20. Wheatley D. Clenbuterol ("Spiropent'): a long-acting bronchodilator. Curr Med Res Opin. 1982;8(2):113-9.
21. Pasotti C, Capra A, Vibelli C. NAB 365 (clenbuterol) and salbutamol in asthmatics: a double-blind clinical trial. Int J Clin Pharmacol Biopharm. 1979;17(4):176-80.
22. Baronti A, Grieco A, Vibelli C. Oral NAB 365 (clenbuterol) and terbutaline in chronic obstructive lung disease: a double-blind, two-week study. Int J Clin Pharmacol Ther Toxicol. 1980;18(1):21-5.
23. Salorinne Y, Stenius B, Tukiainen P, Poppius H. Double-blind cross-over comparison of clenbuterol and salbutamol tablets in asthmatic out-patients. Eur J Clin Pharmacol. 1975;8(3-4):189-95.
24. Al-Majed AA, Khalil NY, Khbrani I, Abdel-Aziz HA. Clenbuterol Hydrochloride. Profiles Drug Subst Excip Relat Methodol. 2017;42:91-123.
25. Bergstrom J, Hermansen L, Hultman E, Saltin B. Diet, muscle glycogen and physical performance. Acta Physiol Scand. 1967;71(2):140-50.
26. DeFronzo RA, Tobin JD, Andres R. Glucose clamp technique: a method for quantifying insulin secretion and resistance. Am J Physiol. 1979;237(3):E214-23.
27. Ngala RA, O'Dowd J, Wang SJ, Agarwal A, Stocker C, Cawthorne MA, et al. Metabolic responses to BRL37344 and clenbuterol in soleus muscle and C2C12 cells via different atypical pharmacologies and beta2-adrenoceptor mechanisms. Br J Pharmacol. 2008;155(3):395-406.
28. Ngala RA, O'Dowd J, Wang SJ, Stocker C, Cawthorne MA, Arch JR. Beta2-adrenoceptors and non-beta-adrenoceptors mediate effects of BRL37344 and clenbuterol on glucose uptake in soleus muscle: studies using knockout mice. Br J Pharmacol. 2009;158(7):1676-82.
29. Nevzorova J, Bengtsson T, Evans BA, Summers RJ. Characterization of the beta-adrenoceptor subtype involved in mediation of glucose transport in L6 cells. Br J Pharmacol. 2002;137(1):9-18.
30. Nevzorova J, Evans BA, Bengtsson T, Summers RJ. Multiple signalling pathways involved in beta2-adrenoceptor-mediated glucose uptake in rat skeletal muscle cells. Br J Pharmacol. 2006;147(4):446-54.
31. Minneman KP, Pittman RN, Molinoff PB. Beta-adrenergic receptor subtypes: properties, distribution, and regulation. Annu Rev Neurosci. 1981;4:419-61.
32. Kawai Y, Powell A, Arinze IJ. Adrenergic receptors in human liver plasma membranes: predominance of beta 2- and alpha 1-receptor subtypes. J Clin Endocrinol Metab. 1986;62(5):827-32.
