# Supplementary material for: Effect of β2-agonist treatment on insulin-stimulated peripheral glucose disposal in healthy men in a randomised placebo-controlled trial
Source: Nat Commun. 2023 Jan 12;14:173. doi: 10.1038/s41467-023-35798-5 (PMC9835033; doi:10.1038/s41467-023-35798-5)
Supplement: Supplementary file 3 — Reporting Summary [file 41467_2023_35798_MOESM3_ESM.pdf]

nature portfolio

Corresponding author(s): Joris Hoeks

Last updated by author(s): 16-12-2022



# Reporting Summary

Nature Portfolio wishes to improve the reproducibility of the work that we publish. This form provides structure for consistency and transparency in reporting. For further information on Nature Portfolio policies, see our Editorial Policies and the Editorial Policy Checklist.

## Statistics

For all statistical analyses, confirm that the following items are present in the figure legend, table legend, main text, or Methods section.

| n/a | Confirmed | |
|---|---|---|
| ☐ | ☒ | The exact sample size (*n*) for each experimental group/condition, given as a discrete number and unit of measurement |
| ☒ | ☐ | A statement on whether measurements were taken from distinct samples or whether the same sample was measured repeatedly |
| ☐ | ☒ | The statistical test(s) used AND whether they are one- or two-sided<br>*Only common tests should be described solely by name; describe more complex techniques in the Methods section.* |
| ☒ | ☐ | A description of all covariates tested |
| ☐ | ☒ | A description of any assumptions or corrections, such as tests of normality and adjustment for multiple comparisons |
| ☐ | ☒ | A full description of the statistical parameters including central tendency (e.g. means) or other basic estimates (e.g. regression coefficient) AND variation (e.g. standard deviation) or associated estimates of uncertainty (e.g. confidence intervals) |
| ☒ | ☐ | For null hypothesis testing, the test statistic (e.g. *F*, *t*, *r*) with confidence intervals, effect sizes, degrees of freedom and *P* value noted<br>*Give P values as exact values whenever suitable.* |
| ☒ | ☐ | For Bayesian analysis, information on the choice of priors and Markov chain Monte Carlo settings |
| ☒ | ☐ | For hierarchical and complex designs, identification of the appropriate level for tests and full reporting of outcomes |
| ☒ | ☐ | Estimates of effect sizes (e.g. Cohen's *d*, Pearson's *r*), indicating how they were calculated |

*Our web collection on statistics for biologists contains articles on many of the points above.*

## Software and code

Policy information about availability of computer code

| | |
|---|---|
| Data collection | No software was used for the collection of data. |
| Data analysis | Data was analysed using IBM SPSS version 27. Graphs were created using Graphpad Prism version 9.3.1. |

For manuscripts utilizing custom algorithms or software that are central to the research but not yet described in published literature, software must be made available to editors and reviewers. We strongly encourage code deposition in a community repository (e.g. GitHub). See the Nature Portfolio guidelines for submitting code & software for further information.

## Data

Policy information about availability of data

All manuscripts must include a data availability statement. This statement should provide the following information, where applicable:

- Accession codes, unique identifiers, or web links for publicly available datasets
- A description of any restrictions on data availability
- For clinical datasets or third party data, please ensure that the statement adheres to our policy

Source data for all tables and figures can be found in the Source Data file immediately following publication with no end date. The study protocol can be found in the supplementary information (Supplementary Note 1). For academic purposes, the de-identified and processed participant data can be requested from the corresponding author (J.hoeks@maastrichtuniversity.nl) with no end date, following the completion of a signed data access agreement form.

## Human research participants

Policy information about studies involving human research participants and Sex and Gender in Research.

| | |
|---|---|
| Reporting on sex and gender | In the study, only male participants were included. |
| Population characteristics | Healthy young males aged between 18-30 years with a BMI between 20-25 kg/m2 and who did not participate in organised or structured physical exercise. |
| Recruitment | Subjects will be recruited in Maastricht and surroundings by means of posters and advertisements in local newspapers and online . Also, subjects that previously participated in other studies at the Department of Nutrition and Movement Sciences at Maastricht University or were not eligible to participate in other studies will be approached by means of a standardized email providing a short explanation of the study design. This type of recruitment will only occur if subjects indicated that they want to be approached for participation in future studies (signed consent) and appear to fulfill the inclusion criteria. The researchers will contact individuals who are interested in participating in the study by telephone, only after the individual has sought contact with the researcher on his or her accord first. By contacting responders by telephone first, the burden of travel and time effort is reduced for the potential subject and researchers. In the telephone interview, the goal of the study will be explained, and the basic inclusion criteria will be discussed. When responders are interested, they will receive detailed subject information via e-mail or mail accompanied by a general brochure (provided by the Dutch government) about participating in a medical study. They will be instructed to read this information carefully and to ask questions if things are unclear. The researcher will contact the possible participant again at least 7 days after the study information was received by the participant. If responders want to participate after reading the study information and seem to be eligible, they are invited for a screening.<br>Participants received a compensation of 500 euros for completion of the study. |
| Ethics oversight | The study was reviewed and approved by the Ethics Committee of the Maastricht University Medical Centre+ (NL67646.068.18) |

Note that full information on the approval of the study protocol must also be provided in the manuscript.

# Field-specific reporting

Please select the one below that is the best fit for your research. If you are not sure, read the appropriate sections before making your selection.

☒ Life sciences ☐ Behavioural & social sciences ☐ Ecological, evolutionary & environmental sciences

For a reference copy of the document with all sections, see nature.com/documents/nr-reporting-summary-flat.pdf

# Life sciences study design

All studies must disclose on these points even when the disclosure is negative.

| | |
|---|---|
| Sample size | Based on a paired samples T-test, the expected standard deviation in glucose disposal during a clamp (Rd) in young, lean subjects (9.7 µmol/kg/min) and an expected mean difference of 25%, which is in our opinion a physiological relevant percentage, we calculated that 11 subjects would be required to reject the null hypothesis with a probability (power) of 80%. The type 1 error probability (a) is 0.05. |
| Data exclusions | For the acute effects of clenbuterol, indirect calorimetry data for one subject was excluded due to a technical error. For one subject, the muscle biopsy could not be obtained due to technical reasons. Muscle tissue from another subject was of inadequate quality for immuno-histochemical analyses. |
| Replication | No measurement were repeated due to the invasive nature of the study measurements. |
| Randomization | Subjects were randomly allocated to the cross-over research design, in a way that the order in which the medication was given (i.e. clenbuterol – placebo and placebo – clenbuterol) occured equally frequent in the entire subject group. |
| Blinding | The study had a double-blinded design. |

# Reporting for specific materials, systems and methods

We require information from authors about some types of materials, experimental systems and methods used in many studies. Here, indicate whether each material, system or method listed is relevant to your study. If you are not sure if a list item applies to your research, read the appropriate section before selecting a response.

## Materials & experimental systems

| n/a | Involved in the study | |
|---|---|---|
| ☐ | ☒ | Antibodies |
| ☒ | ☐ | Eukaryotic cell lines |
| ☒ | ☐ | Palaeontology and archaeology |
| ☒ | ☐ | Animals and other organisms |
| ☐ | ☒ | Clinical data |
| ☒ | ☐ | Dual use research of concern |

## Methods

| n/a | Involved in the study | |
|---|---|---|
| ☒ | ☐ | ChIP-seq |
| ☒ | ☐ | Flow cytometry |
| ☒ | ☐ | MRI-based neuroimaging |

# Antibodies

Antibodies used

GLUT4 (1:50; ab33780; Abcam) (immunohistochemical staining)
caveolin (1:25; 610421; BD Biosciences, Vianen, The Netherlands) (immunohistochemical staining)
p-mTORC2 S2481 antibody (1:1000, #2974, Cell Signalling, Danvers, MA, USA) (western blot)
p-mTORC1 S2448 antibody (1:1000, #2971, Cell signalling, Danvers, MA, USA) (western blot)
VDAC antibody (1:1000, sc-390336, Santa Cruz Biotechnology, Dallas, Texas, USA) (western blot)
TOMM20 (1:10.000, ab18734, Abcam, Cambridge, UK) (western blot)
OxPhos anitbody cocktail (1:1000, ab110411, Abcam, Cambridge, UK) (western blot)
AlexaFluor555 (1:500; A21428, Invitrogen) (immunohistochemistry)
AlexaFluor488 (1:200; A21121, Invitrogen, Invitrogen) (immunohistochemistry)
IRDye800 conjugated antibodies (Donkey anti-rabbit IRDye800; 1:10.000; 926-32213; Li-COR, or Donkey anti-mouse IRDye800; 1:10.000; 626-32212; Li-COR, Lincoln, Nebraska, USA) (western blot)

Validation

For primary antibodies:
GLUT4: suitable for western blot and immunohistochemistry and validated in rat skeletal muscle.
p-mTORC2 S2481: suitable for western blot. Reactivity to human, mouse, rat and monkey. Validated in 293 cells untreated or treated with 20% FBS for 30 minutes.
p-mTORC1 S2448: suitable for western blot. Reactivity to human, mouse, rat and monkey. Validated in 293 cells untreated or treated with EGF (100 ng/mL).
VDAC: suitable for western blot. Reactivity to human, mouse, and rat. Validated in HL-60, Jurkat, HeLa, and A-431 whole cell lysates, as well as rat and human heart tissue extracts.
TOMM20: Suitable for western blot. Reactivity to mouse, rat and human. Validated for western blot in HepG2 and HeLa whole cell lysate.
OxPhos antibody cocktail: suitable for western blot. Reactivity to human. Validated with isolated mitochondria from MRC5 fibroblasts.

# Clinical data

Policy information about clinical studies
All manuscripts should comply with the ICMJE guidelines for publication of clinical research and a completed CONSORT checklist must be included with all submissions.

Clinical trial registration

The study was registered at ClinicalTrials.gov under the identifier NCT03800290

Study protocol

The full protocol is available in the supplementary information

Data collection

Data were collected between August 13th 2019 and April 23rd 2021 at Maastricht University. The study was temporarily halted from March 16th 2020 until July 1st 2020 due to the national COVID-19 lockdown in The Netherlands.

Outcomes

Primary outcome: Insulin-stimulated peripheral glucose disposal during the high-insulin infusion of the two-step hyperinsulinemic-euglycemic clamp corrected for baseline (delta RD)
Secondary outcome: Skeletal muscle GLUT4 translocation as assessed by means of immunihistochemistry
